# A New Factor LapD Is Required for the Regulation of LpxC Amounts and Lipopolysaccharide Trafficking

**DOI:** 10.3390/ijms23179706

**Published:** 2022-08-26

**Authors:** Alicja Wieczorek, Anna Sendobra, Akshey Maniyeri, Magdalena Sugalska, Gracjana Klein, Satish Raina

**Affiliations:** Laboratory of Bacterial Genetics, Gdansk University of Technology, 80-233 Gdansk, Poland

**Keywords:** lipopolysaccharide, LpxC, cardiolipin synthase A, LPS assembly proteins LapB, LapC (YejM) and LapD, acyltransferases LpxL and LpxM, heptosyltransferase I WaaC, MsbA, trigger factor, MicA sRNA

## Abstract

Lipopolysaccharide (LPS) constitutes the major component of the outer membrane and is essential for bacteria, such as *Escherichia coli*. Recent work has revealed the essential roles of LapB and LapC proteins in regulating LPS amounts; although, if any additional partners are involved is unknown. Examination of proteins co-purifying with LapB identified LapD as a new partner. The purification of LapD reveals that it forms a complex with several proteins involved in LPS and phospholipid biosynthesis, including FtsH-LapA/B and Fab enzymes. Loss of LapD causes a reduction in LpxC amounts and vancomycin sensitivity, which can be restored by mutations that stabilize LpxC (mutations in *lapB*, *ftsH* and *lpxC* genes), revealing that LapD acts upstream of LapB-FtsH in regulating LpxC amounts. Interestingly, LapD absence results in the substantial retention of LPS in the inner membranes and synthetic lethality when either the lauroyl or the myristoyl acyl transferase is absent, which can be overcome by single-amino acid suppressor mutations in LPS flippase MsbA, suggesting LPS translocation defects in Δ*lapD* bacteria. Several genes whose products are involved in cell envelope homeostasis, including *clsA*, *waaC*, *tig* and *micA*, become essential in LapD’s absence. Furthermore, the overproduction of acyl carrier protein AcpP or transcriptional factors DksA, SrrA can overcome certain defects of the LapD-lacking strain.

## 1. Introduction

The most characteristic feature of Gram-negative bacteria, such as *Escherichia coli*, is the presence of an asymmetric outer membrane (OM), which is essential for their viability [1]. This asymmetric nature of OM is critical for endowing a permeability barrier to prevent the entry of bulky toxic molecules inside the cells and is based upon the unique distribution pattern that restricts the presence of lipopolysaccharide (LPS) in the outer leaflet of the cell envelope, with phospholipids facing its inner leaflet [1,2]. LPS comprises the major component of OM, covering nearly 75% of OM, and is the major virulence factor and the causative agent of sepsis due to Gram-negative bacteria [1,3,4]. Although the LPS composition is highly heterogenous, they overall share a common basic structure. Thus, LPS can be divided into three parts, with a highly conserved hydrophobic membrane-anchored lipid A, a core oligosaccharide, to which an oligosaccharide of variable length, called the *O*-antigen, is attached in bacteria with smooth LPS [2,3]. The most conserved part of LPS lipid A constitutes the endotoxin principal and in *E. coli* is composed of a bisphosphorylated β(1→6)-linked GlcN disaccharide, to which generally six asymmetric fatty acids are linked via ester and amide linkages. LPS biosynthesis begins with the acylation of UDP-GlcNAc by LpxA with (*R*)-3-hydroxymyristate derived from (*R*)-3-hydroxymyristoyl-ACP, followed by successive reactions catalyzed by additional enzymes with LpxC-mediated deacylation constituting the first committed step [3,5,6,7,8]. This generates a lipid IV_A_ precursor to which two 3-deoxy-α-d-*manno*-oct-2-ulosonic acid (Kdo) residues are attached by the essential enzyme WaaA, at the reducing GlcN residue [3,9]. This generates the key precursor intermediate, termed Kdo_2_-lipid IV_A_ [3,9]. This precursor species acts as an acceptor for the acylation by LpxL and LpxM generating hexa-acylated Kdo_2_-lipid A, which is further extended by various glycosyltransferases for incorporating different sugar molecules for the completion of core biosynthesis [9,10].

The heterogeneity of LPS composition can arise due to changes in the lipid A acylation, modification of phosphate residues of lipid A by phosphoethanolamine (*P-EtN*), 4-amino-4-deoxy-l-arabinose (l-Ara4N), the non-stoichiometric incorporation of an additional third Kdo residue, uronic acid, rhamnose, modification of the second Kdo residue by phosphoethanolamine, truncation in the outer core and changes in the phosphorylation of the inner core [4,11,12]. This results in the presence of different glycoforms of LPS. This heterogeneity of LPS is regulated by regulon members of the cell envelope-responsive sigma factor RpoE, two-component systems such as BasS/R, PhoP/Q, PhoB/R and Rcs [11]. Thus, specific glycoforms are synthesized when the RpoE regulon is induced due to severe impairment in the cell envelope composition, either due to misfolding of outer membrane proteins (OMPs) or imbalance in their synthesis or when LPS biosynthesis is compromised [11]. These switches in the glycoform synthesis are regulated at the transcriptional level by a specific increase in the expression of certain genes as well as translational repression by sRNAs such as RybB and MgrR [11,13,14]. Although the structural analysis of LPS supports the role of such sRNAs, the physiological significance and molecular basis of specific mRNA:sRNA interactions have not yet been elucidated. The incorporation of some of these non-stoichiometric modifications, such as *P-EtN* and l-Ara4N in the lipid A part, are known to confer resistance to cationic antimicrobial peptides such as polymyxin B and can be important in bacterial adaptation to various host and environmental niches [4].

**Figure 1:**
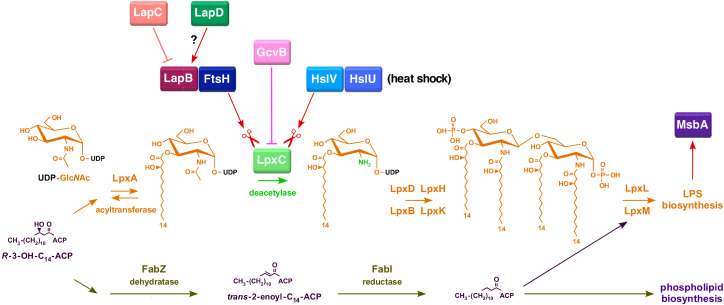
Key steps in the regulation of the first committed step in LPS biosynthesis catalyzed by LpxC and LPS transport mediated by MsbA across the inner membrane. Schematic illustration depicting utilization of the same metabolic precursor (*R*)-3-hydroxymyristate by LpxA and by FabZ in LPS and phospholipid biosynthesis, respectively. As the reaction catalyzed by LpxA is thermodynamically unfavorable, LpxC-mediated deacylation constitutes the first committed step in LPS biosynthesis. LpxC amounts are regulated by its turnover by the FtsH-LapB complex and at high temperature by HslVU protease. LapC acts as an antagonist of LapB to regulate LPS biosynthesis as per its demand. Once LPS is assembled, it is flipped across the inner membrane by MsbA for its further transport. Scissors depict proteolysis by FtsH and HslVU proteases. A newly identified LapD protein that co-purifies with LapB is depicted with a question mark.

The viability of all Gram-negative bacteria, including *E. coli*, requires a tight balance between phospholipids and LPS amounts, which is held at a constant ratio of (1:0.15) for the maintenance of outer membrane asymmetry [7]. This is achieved by the regulated turnover of LpxC via its proteolytic control and the activity of the FabZ dehydratase enzyme [15]. The regulation of LpxC amounts is critical as it mediates the first committed step in LPS biosynthesis, while FabZ initiates phospholipid biosynthesis [15,16,17,18]. Since these two essential enzymatic pathways use the same (*R*)-3-hydroxymyristate as the common metabolic precursor, its depletion due to diversion in either pathway is toxic for bacteria, and hence either excess or reduced amounts of LPS are lethal for bacteria [15,17]. The stability of LpxC and, in turn, LpxC amounts are regulated in a complex manner with several pathways involved in adjusting these amounts as per the cellular demand of LPS and also depends on the composition of fatty acids [2,17,18,19]. However, the molecular basis of alteration of the in vivo stability of LpxC and its amounts are not fully understood (Figure 1). One of the key enzymes that participate in the proteolysis of LpxC is the essential inner membrane (IM)-anchored ATP-dependent FtsH metalloprotease [15]. This degradation of LpxC by FtsH requires another essential factor called the LPS assembly protein LapB [17,20]. Thus, a deletion of either the *lapB* gene or the *ftsH* gene is lethal due to the stabilization of LpxC, which results in a toxic increase in the LPS synthesis [17]. However, this FtsH-LapB-mediated proteolysis can be counteracted by another essential protein designated LapC (previously YejM) [21,22,23,24,25,26]. The *lapC* gene was identified since a mutation in its coding sequence that causes truncation in the LapC’s periplasmic domain could allow the deletion of the essential *lapB* gene [21]. These genetic studies suggested that LapC antagonizes LapB-FtsH-mediated proteolysis [21,23]. Consistent with such a role for LapC, the truncation of its non-essential periplasmic domain or the depletion of *lapC* causes increased LpxC degradation, resulting in a concomitant reduction in LPS and LpxC amounts [21,23,25]. Furthermore, LapB and LapC co-purify, and both bind LPS [21,22]. However, how LapC and LapB adjust the rate of LpxC degradation is not understood (Figure 1). It is also not known if the lethality due to the excessive synthesis of LPS in *ftsH* and *lapB* mutants is due to the depletion of acyl-ACP pools or due to the retention of LPS in the IM and its poor translocation to the OM. Similarly, the physiological factors, other than increased LpxC degradation, which cause the lethality in the absence of LapC, are not identified. To add to this complexity, LpxC can also be degraded in vivo in an FtsH-LapB independent manner by the HslVU protease complex, and this degradation could be more relevant at high temperatures [21].

LPS assembly further requires efficient LPS translocation with the first step of its flipping across the IM mediated by the essential ATP-dependent transporter MsbA [27]. In the subsequent steps, LPS is translocated to the OM by another essential transenvelope machinery, comprising seven proteins that span all three compartments of the cell [28]. MsbA uses its hydrocarbon ruler properties to prevent or reduce the translocation of underacylated LPS species [29,30,31,32]. This preferential selectivity for hexa-acylated lipid A provides an essential checkpoint, ensuring only mature LPS is translocated to the OM [2]. Thus, not surprisingly, suppressor mutations that overcome the lethality of either Δ(*lpxL lpxP lpxM*) or Δ*waaA* strains synthesizing lipid IV_A_ derivatives map to the *msbA* gene, presumably by relaxing the selectivity of MsbA for the translocation of underacylated LPS [9,32]. In the translocation of underacylated LPS, MsbA is aided by cardiolipins [32,33]. Consistent with such a requirement for cardiolipins, mutational combinations of Δ(*clsA msbA*), Δ(*clsA lpxL*) and Δ(*clsA waaA*) are lethal, which can be overcome by suppressor mutations in the *msbA* gene [32]. However, the molecular basis of such lethality and how cardiolipins aid MsbA in LPS transport remains unknown.

**Figure 2:**
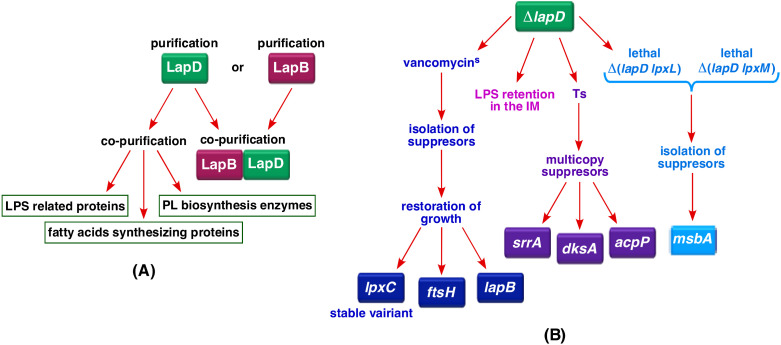
Schematic depiction of various approaches that identify LapD as a regulator of LPS biosynthesis regulating LpxC amounts and assisting MsbA in the LPS transport. Identification of LapD as an IM protein associated with LapB and co-purification of LapD with proteins involved in LPS/phospholipid biosynthesis (**A**). Suppressors of vancomycin sensitivity of Δ*lapD* and of various synthetic lethal combinations identify suppressor mutations either in genes that stabilize LpxC or in the *msbA* gene, revealing that LapD acts upstream of LapB and aids MsbA-mediated LPS transport (**B**).

To further understand the balanced regulation of LPS and phospholipid biosynthesis, we first carefully examined proteins that interact with LapB to identify if any factors had been previously missed. This analysis identified an additional protein YhcB, designated LapD, which co-purifies with LapA and LapB proteins (Figure 2A). This co-purification was also validated when the purification profile of LapD was analyzed, which showed that LapD co-purifies not only with LPS assembly proteins but also with several proteins involved in either LPS transport or its biosynthesis or the fatty acid synthesis (Figure 2A). We have previously shown that the *lapD* gene is required for the growth of *E. coli* at critical high temperatures [34]. LapD (YhcB) is an inner membrane protein and has recently been implicated in either the cell division process or the cell envelope homeostasis; although, molecular mechanisms in either of these functions remain unknown [35,36,37,38]. In this work, we show that in the absence of LapD, LpxL and LpxM acyl transferases become essential and the synthetic lethality of either Δ(*lpxL lapD*) or Δ(*lpxM lapD*) can be overcome by extragenic suppressor mutations mapping to the essential *msbA* gene (Figure 2B). We further show that Δ*lapD* strains exhibit sensitivity to antibiotics such as vancomycin and reduced amounts of LpxC. Consistent with a role in the regulation of LPS amounts and the interaction with LapB protein, mutations that either render LpxC resistant to FtsH-mediated proteolysis or loss-of-function variants in the *lapB* gene can overcome the sensitivity of Δ*lapD* bacteria to vancomycin (Figure 2B). Consistent with a role in these essential processes, various growth defects of a Δ*lapD* derivative can be overcome when the acyl carrier protein AcpP is overproduced. Since the AcpP protein acts as a key component in the fatty acid synthesis pathway and interacts with various acyl transferases involved in the biosynthesis of lipid A and the phospholipid synthesis [39], its identification as a multicopy suppressor of Δ*lapD* defects is consistent with a critical role in balanced biosynthesis of LPS and phospholipids. We present genetic and biochemical data supporting a role for LapD acting upstream of LapB by acting in an antagonistic manner, thereby controlling LpxC levels and could also aid MsbA-mediated LPS translocation.

## 2. Results

### 2.1. LapD Is Part of LapA/LapB Complex and Co-Purifies with Several Proteins Involved in LPS and Phospholipid Biosynthesis

**Figure 3:**
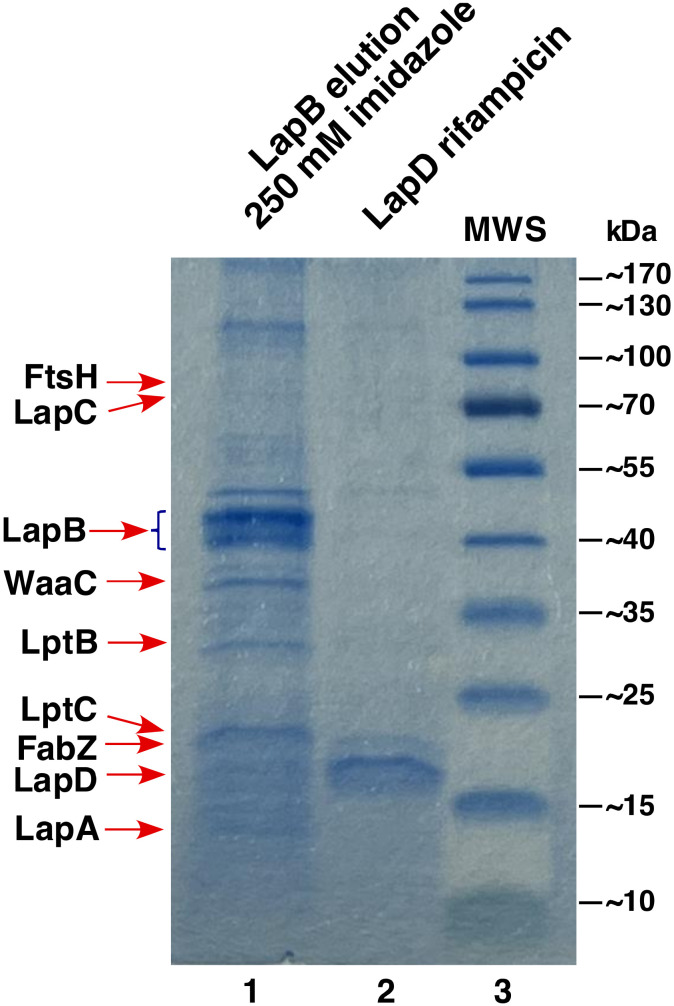
LapD as a new interacting partner of LapB. Purification profile of His_6_-tagged LapB protein from the IM fraction after elution with 250 mM imidazole. Lane 1 shows co-purifying proteins with LapB that include LapD. All major co-purifying proteins are indicated by arrows. In lane 2, purified LapD protein was applied. Proteins were resolved on a 12% SDS-PAGE, stained by Coomassie Brilliant Blue. Lane 3 shows pre-stained molecular weight standards.

Examination of proteins that co-purify with LapB revealed the presence of a new component designated LapD in addition to previously known interacting partners such as LapA, FtsH, WaaC, FabZ and Lpt proteins (Figure 3). MALDI-TOF analysis identified peptides QQQALQYELEK, SAELLDTMAHDYR, SSSSLLPELSAEANPFR and LAESEASNDQAPVQMPRDYSEGASGLLR covering more than 52% of the entire LapD amino acid sequence. We had previously identified the *lapD* (*yhcB*) gene in a global screen of *E. coli* genomic knockouts, whose products are required for growth at high temperature [34]. Besides the temperature-sensitive (Ts) phenotype, Δ*lapD* bacteria are also sensitive to antibiotics such as vancomycin, suggesting defects in the OM barrier function (see below). We also had observed earlier that the deletion of the *lapD* gene cannot be tolerated in a strain devoid of six cytoplasmic peptidyl-prolyl *cis*/*trans* isomerases [40]. However, the molecular basis of such a lethality remained unknown. Although LapD has recently been implicated in cell division or the maintenance of cell envelope homeostasis, its function has remained unknown [36,37].

**Figure 4:**
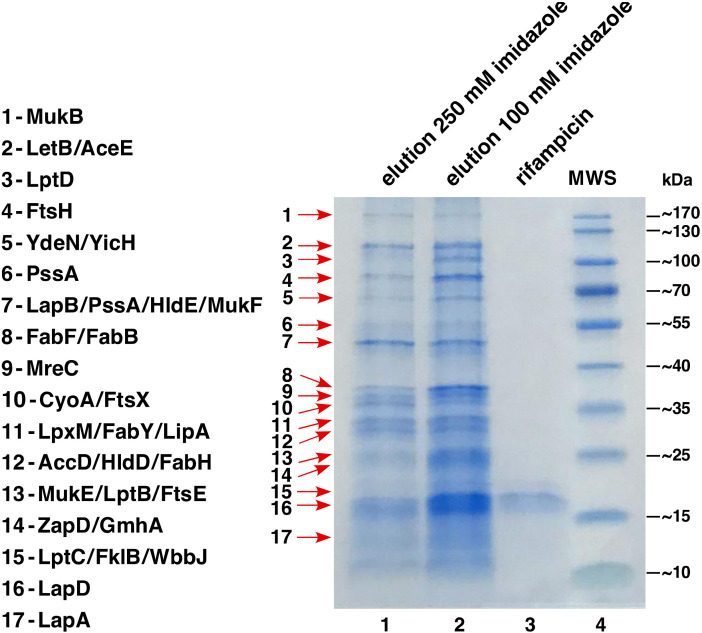
LapD forms a complex in the IM with proteins involved in LPS and phospholipid biosynthesis. Purification profile of His_6_-tagged LapD protein from the IM fraction after elution with 250 mM and 100 mM imidazole (lanes 1 and 2, respectively). Lane 3 shows the migration of His_6_-tagged LapD protein obtained after rifampicin treatment during the induction of its synthesis. All major co-purifying proteins are indicated by arrows. Proteins were resolved on a 12% SDS-PAGE, stained by Coomassie Brilliant Blue. Lane 4 shows pre-stained molecular weight standards.

To elucidate LapD function, a His_6_-tagged derivative was purified from IM fractions and co-eluted proteins identified by MALDI-TOF to reveal its interacting partners (Figure 4). These experiments showed that the majority of proteins that co-purify with LapD are involved in either LPS biosynthesis/assembly or transport, which include (LpxM, FtsH, HldE, HldD, GmhA, WbbJ, LapA/LapB and LptB/C/D) and phospholipid/fatty acid biosynthesis (PssA, AccD and FabB/F/H/Y). A few proteins involved in cell shape and chromosomal segregation (MukB/F/E, MreC and ZapD) were also identified in such pull-down experiments (Figure 4). In addition, a cytoplasmic peptidyl-prolyl *cis*/*trans* isomerase FklB, belonging to the family of FK506-binding proteins, was identified among co-eluted proteins (Figure 4). Among co-eluting proteins, LpxM adds the last acyl chain to complete the synthesis of hexa-acylated lipid A after the addition of two Kdo residues [41], while FtsH is the essential IM protease, one of whose substrates is LpxC [15]. Other prominent co-purifying enzymes with LapD are involved in phospholipid biosynthesis. Thus, besides fatty acid biosynthetic Fab enzymes, PssA (phosphatidylserine synthase) mediates the first committed step for phosphatidylethanolamine biosynthesis [42]. These results demonstrate that LapD forms a complex in the IM with proteins involved in LPS assembly, its biogenesis and transport (LapA/B, LpxM and Lpt), and phospholipid biosynthesis.

### 2.2. LapD Is Required to Maintain Levels of LpxC

**Figure 5:**
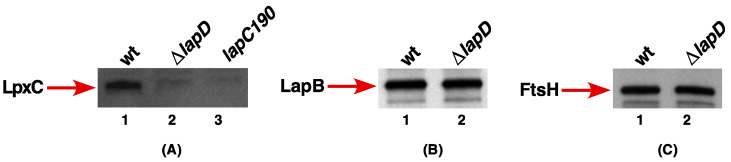
An absence of LapD causes a reduction in the amounts of LpxC. An immunoblot of whole cell lysates obtained from isogenic strains with indicated genotypes using LpxC-specific antibodies (**A**). In parallel, samples from the wild type and Δ*lapD* were immunoblotted with LapB-specific antibodies (**B**) and with FtsH-specific antibodies (**C**). An equivalent amount of total proteins was resolved by SDS-PAGE prior to immunoblotting.

To further investigate the function of LapD and its requirement in the regulation of LPS, we analyzed the levels of the LpxC enzyme. Isogenic bacterial cultures of the wild type and a Δ*lapD* strain were grown at 30 °C (permissive growth conditions) and then shifted to 43 °C for 2 h. Such bacterial cultures were used to prepare whole cell lysates. As a control, we also included a previously well-characterized isogenic *lapC190* bacterial strain, which lacks the periplasmic domain of LapC and exhibits diminished amounts of LpxC. The equivalent amounts of total proteins were resolved on a 12% SDS-PAGE, and LpxC amounts were analyzed by immunoblotting using LpxC-specific antibodies. Such experiments revealed that under such growth conditions, Δ*lapD* bacteria have reduced amounts of LpxC (Figure 5A, lane 2). This is consistent with previous results, where *lapC190* mutant bacteria also exhibit reduced amounts of LpxC (Figure 5A, lane 3). Thus, *lapC190* and Δ*lapD* bacteria both have reduced amounts of LpxC in contrast to the elevated levels of LpxC in *lapB* bacteria. As a control, we also estimated the amounts of LapB and FtsH in whole cell lysates obtained from the isogenic wild type and its Δ*lapD* derivative by immunoblotting with LapB- and FtsH-specific antibodies (Figure 5B,C). As can be seen, no major differences in LapB and FtsH amounts were observed, in contrast to a reduction in LpxC amounts in Δ*lapD* bacteria. Our results showing a reduction in LpxC amounts can explain phenotypic defects such as the loss of permeability, reflected in the sensitivity to antibiotics such as vancomycin when LapD is absent.

### 2.3. Suppressor Mutations That Stabilize LpxC Can Restore the Wild-Type-like Growth of ΔlapD Bacteria on Vancomycin-Supplemented Growth Medium

**Figure 6:**
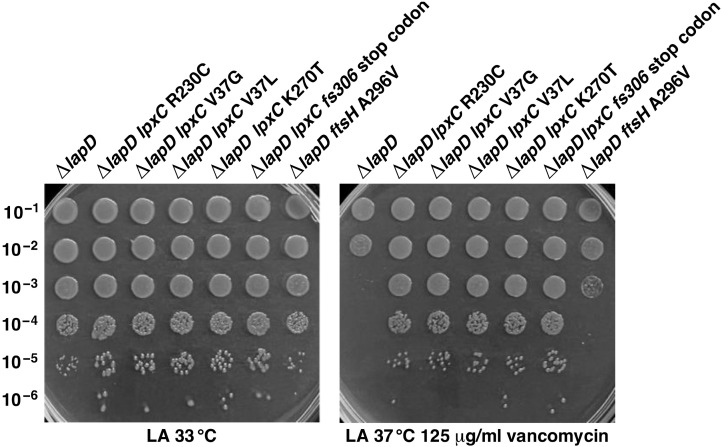
Mutations that stabilize LpxC suppress vancomycin sensitivity of Δ*lapD* bacteria. Growth of isogenic cultures of strains with Δ*lapD* and with suppressor mutations in the *lpxC* gene was quantified by spot dilution on LA with and without supplementation of vancomycin. The relevant genotype and temperature of incubation are indicated.

In previous work, we showed that a Ts phenotype and reduced levels of LpxC in *lapC190* bacteria can be rescued by single amino acid suppressor mutations mapping to *lpxC*, *ftsH* and *lapA*/*lapB* operon [21]. This suppression of *lapC190* mutant bacteria by such extragenic suppressors was explained on the basis of increasing LpxC amounts. Thus, we reasoned that the introduction of such extragenic suppressor mutations that stabilize LpxC should also suppress growth defects of Δ*lapD* bacteria. To achieve this, previous *lapC190*::cm^R^ strains with suppressor mutations in either the *lpxC* gene or the *ftsH* gene [21] were first used as recipients to remove the *lapC190* mutation by the introduction of tightly linked *napA*::Tn*10* scoring for the loss of Cm resistant marker to have only an *lpxC* or *ftsH* chromosomal mutation. Thus, bacteria with a wild-type copy of the *lapC* gene but with chromosomal *lpxC* single amino acid substitutions or a frameshift that render LpxC resistant to proteolysis (SR23812 *lpxC* R230C, SR23814 *lpxC* V37G, SR23816 *lpxC* V37L, SR23818 *lpxC* K270T, and SR23820 *lpxC fs306* stop codon) and the strain SR23822 with *ftsH* A296V served as recipients (see Section 4.1). Into such *lpxC* and *ftsH* variants, the Δ*lapD* mutation was introduced by bacteriophage P1-mediated transductions and analyzed for restoration of resistance to vancomycin. All such strains with the deletion of the *lapD* gene were found to be resistant to vancomycin, unlike isogenic Δ*lapD* bacteria, which are sensitive (Figure 6). However, the restoration of growth of Δ*lapD* with *ftsH* A296V mutation on vancomycin was somewhat lower than when stable *lpxC* variants were introduced (Figure 6). In later sections (see Section 2.10), we have again verified that the above-mentioned mutations in the *lpxC* gene lead to increased accumulation of LpxC. Thus, we can conclude that a restoration of LpxC stability by introducing LpxC stable variants in Δ*lapD* bacteria can overcome membrane permeability defects.

### 2.4. Suppressor Mutations in the lapB Gene That Prevent LpxC Degradation and Restore the Growth of lapC190 Mutant Bacteria Can Also Restore the Wild-Type-like Growth of ΔlapD Bacteria on Vancomycin-Supplemented Growth Medium

**Figure 7:**
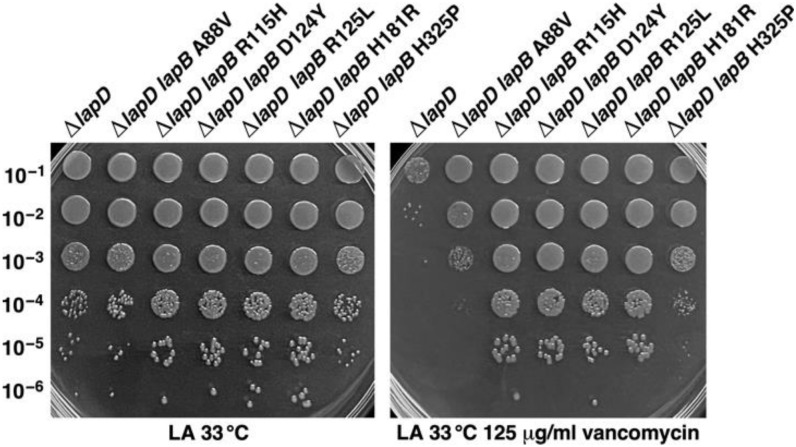
Well-characterized suppressor mutations in the *lapB* gene that prevent LpxC proteolysis suppress vancomycin sensitivity of Δ*lapD* bacteria. Growth of isogenic cultures of strains with Δ*lapD* and with suppressor mutations in the *lapB* gene was quantified by spot dilution on LA with and without supplementation of vancomycin. The relevant genotype and temperature of incubation are indicated.

We previously isolated several suppressor mutations that overcome Ts and permeability defects of *lapC190* mutant bacteria mapping to the *lapB* gene [21]. Most of such suppressor mutations had severely reduced LapB amounts, which in turn prevented LpxC degradation [21]. As Δ*lapD* bacteria have reduced LpxC quite like *lapC190* bacteria, we reasoned that the introduction of such *lapB* mutations should also restore the growth of a Δ*lapD* strain under conditions such as exposure to vancomycin. Thus, as described in the above section, firstly the *lapC190* mutation was removed by introducing *napA*::Tn*10*, selecting for the loss of the Cm^R^ cassette that replaces the periplasmic domain of LapC to have only a single amino acid *lapB* suppressor mutation on the chromosome. Such isogenic strains with an intact copy of the *lapC* gene served as a recipient to bring in the *lapD* deletion. This resulted in generating strains SR23857 (*lapB* H325P Δ*lapD*), SR23859 (*lapB* A88V Δ*lapD*), SR23861 (*lapB* H181R Δ*lapD*), SR23863 (*lapB* R115H Δ*lapD*), SR23865 (*lapB* D124Y Δ*lapD*) and SR23867 (*lapB* R125L Δ*lapD*) (see Methods section). Such isogenic strains along with parental Δ*lapD* were tested for the growth at permissive growth conditions and when growth medium was supplemented with vancomycin by spot dilution assay. Such experiments reveal that single amino acid substitutions in the *lapB* gene, which render LpxC stable, can confer vancomycin resistance to Δ*lapD* bacteria, although to a different extent (Figure 7). Among the tested *lapB* mutants, the introduction of *lapB* R115H, *lapB* D124Y, *lapB* R125L and *lapB* H181R in Δ*lapD* bacteria, conferred better suppression in terms of restoration of the growth on vancomycin-supplemented growth medium (Figure 7). Thus, we can conclude that the reduction in LpxC amounts in Δ*lapD* bacteria can be compensated when LpxC is stabilized by introducing loss-of-function mutations in the *lapB* gene in a manner similar to that previously observed with a *lapC190* mutant strain. Hence, quite like LapC, LapD could function upstream of LapB and act as its antagonist to prevent excessive degradation of LpxC. However, it should be noted that the Ts phenotype of the Δ*lapD* derivative is not fully suppressed by mutations in the *lapB* gene, which is not the case with *lapC190* mutant bacteria [21].

### 2.5. Reduction in the LPS Synthesis Is Lethal for ΔlapD Bacteria

If indeed, LapD regulates LpxC proteolysis in a manner antagonistic to LapB and acts in a pathway similar to LapC upstream of LapB to regulate LPS biosynthesis, any reduction in LPS biosynthesis should be toxic to Δ*lapD* bacteria. It should be noted that LapB becomes dispensable when LpxC/LPS amounts are reduced, as shown earlier, when the LPS synthesis is dampened in the presence of dysfunctional LapC or by introducing the *lpxA2*(ts) mutation [17]. Thus, we performed parallel transductions in SM101 *lpxA2*(ts), MN7 *lpxB1*(ts) and GK6075 (*lapC190*) bacteria by introducing a Δ*lapD* mutation, using appropriate controls (Table 1). It is known that SM101 *lpxA2*(ts), MN7 *lpxB1*(ts) and GK6075 (*lapC190*) have reduced amounts of LPS [15,17]. Most significantly, Δ*lapD* could not be introduced in the strains with mutations in either the *lpxA* gene or the *lpxB* gene or the *lapC* gene, while it could be introduced in the wild-type strain (Table 1). In contrast, a *lapB* deletion is readily accepted in *lpxA2*(ts), *lpxB1*(ts) and *lapC190* mutant bacteria, consistent with our earlier results [17,21]. Thus, mutations in genes that cause a reduction in the LPS synthesis are lethal in a Δ*lapD* background and, in converse, the reduction in the LPS synthesis bypasses the lethality associated with Δ*lapB*. These results support the notion that LapD acts upstream of LapB, acting antagonistically, and has a function similar to LapC.

### 2.6. LapD Is Essential When Either LpxL or LpxM Late Acyl Transferase Is Absent and the Conditional Lethality When Cardiolipin Synthase A or WaaC Heptosyl Transferase Is Absent

Data presented from several above-described experiments suggest physical (co-purification) or genetic interaction of LapD with several enzymes involved in LPS assembly or biosynthesis. To further investigate any specific requirement for LapD in these pathways, a series of transductions were performed using strains with a defined individual null mutation in otherwise non-essential genes whose products are known to be involved in either LPS or phospholipid biosynthesis. In the biosynthesis of hexa-acylated lipid A, only *lpxL* and *lpxM* are non-essential genes; although, a deletion of the *lpxL* gene confers a Ts phenotype above 33 °C [43]. Thus, we attempted to construct Δ(*lpxL lapD*) and Δ(*lpxM lapD*) strains using bacteriophage P1-mediated transductions at 30 °C (Table 2). No viable transductants were observed and only when plated in large numbers a few suppressors were obtained (see below).

**Figure 8:**
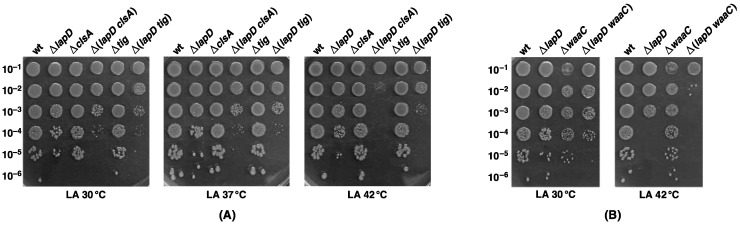
The conditional synthetic lethal phenotype of various mutational combinations reflecting the essentiality of the *lapD* gene when either the LPS is truncated or cardiolipin biosynthesis is disrupted or when the PPIase activity is impaired. Growth of isogenic cultures of strains of wild type, Δ*lapD*, Δ*clsA*, Δ*waaC*, Δ*tig* and various null combinations was quantified by spot dilution on LA at different temperatures (Panels (**A**,**B**)). The relevant genotype and temperature of incubation are indicated.

After the minimal Kdo_2_-lipid A LPS is synthesized, it becomes an acceptor for the incorporation of various sugars, with WaaC being the first enzyme mediating the transfer of the first heptose to the Kdo moiety. Thus, among various transductional combinations, Δ(*waaC lapD*) was constructed and analyzed for growth properties. Next, we examined the requirement of cardiolipins in the absence of LapD. In cardiolipin biosynthesis, ClsA is the main contributor [44,45]. Thus, Δ(*clsA lapD*) strains were also constructed and analyzed further (Table 2). Although Δ(*waaC lapD*) and Δ(*clsA lapD*) viable transductants were obtained at 30 °C, their colony size was smaller than that of the parental strains (Table 2). To quantify growth defects, panels of such strains were examined by spot-dilution assay at different temperatures. As shown, Δ(*clsA lapD*) bacteria form small-sized colonies at 30 and 37 °C, with a reduction of nearly 10^3^ in terms of colony forming units (cfu) (Figure 8A). At 42 °C, such bacteria exhibit a Ts phenotype, conditions under which Δ*lapD* and Δ*clsA* bacteria do not exhibit any major growth defects (Figure 8A). Regarding the growth properties of Δ(*waaC lapD*) bacteria, spot-dilution assays were performed at 30 °C and 42 °C. Even at 30 °C, Δ(*waaC lapD*) bacteria showed a 100-fold reduction in cfu (Figure 8B). At 42 °C, the Δ(*waaC lapD*) combination turns out to be lethal, which is permissive for the growth of either Δ*waaC* or Δ*lapD* strains (Figure 8B). Thus, LapD is essential for the growth of *E. coli* when LPS is either underacylated or when bacteria synthesize the minimal LPS structure composed of Kdo_2_-lipid A due to a lack of WaaC heptosyltransferase. LapD is also critically required for bacterial viability when cardiolipin biosynthesis is compromised, as shown by the conditional synthetic lethality of Δ(*clsA lapD*).

### 2.7. Single Amino Acid Suppressor Mutations in the msbA Gene Can Bypass the Lethality of Δ(lpxL lapD) and Δ(lpxM lapD) Bacteria

**Figure 9:**
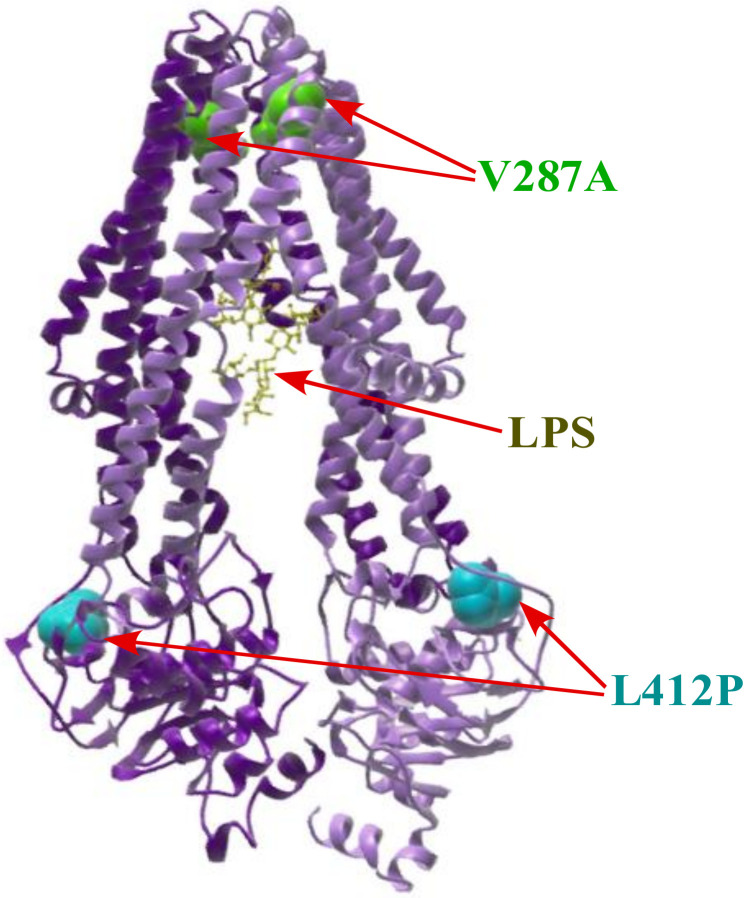
Mutations mapping to the *msbA* gene can bypass the synthetic lethality of Δ(*lpxL lapD*) and Δ(*lpxM lapD*) bacteria. Positions of various single amino acid substitutions in the structure of MsbA (PDB 6BPL) [30] are shown by arrows. The position of the LPS is also indicated and marked by the arrow.

To further understand the molecular basis of the lethality of Δ(*lpxL lapD*) and Δ(*lpxM lapD*) combinations, we sought to isolate extragenic chromosomal suppressor mutations that can overcome this lethal phenotype. Thus, several rounds of bacteriophage P1-mediated transductions were performed by bringing in the null mutation of the *lapD* gene in defined Δ*lpxL* and Δ*lpxM* strains. As shown above, LpxL and LpxM are essential in the absence of LapD. Transductants were plated at 30 °C and few survivors could be obtained. Out of these, two such strains, SR23684 Δ(*lpxL lapD*) sup* and SR23685 Δ(*lpxM lapD*) sup*, were retained for further analysis. To identify the suppressor mutation, we PCR amplified coding regions of *lpxC*, *lapA*/*B*, *lapC*, *ftsH*, *fabZ* and *msbA* genes using the chromosomal DNA of SR23684 and SR23685 as templates. DNA sequencing analysis showed that SR23684 has a single nucleotide change in the codon C**T**G to C**C**G, resulting in a single amino acid exchange of L412P in the *msbA* gene. SR23685 was found to have also a single amino acid exchange of V287A due to the mutation of codon G**T**T to G**C**T. These two independent single amino substitutions in the MsbA structure show that the L412P substitution is in the nucleotide-biding domain and V287A is predicted to be located in the LPS-binding domain (Figure 9). Interestingly, we had recently isolated the *msbA* V287A mutation as a suppressor mutation that restored the growth of the Δ(*lpxM clsA*) derivative [32]. To ensure SR23684 and SR23685 do not carry an additional mutation, the replacement of the *msbA* suppressor by a wild-type copy did not allow restoration of growth using a linked marker in transductions. Isolation of suppressor mutations that overcome the synthetic lethality of Δ(*lpxL lapD*) and Δ(*lpxM lapD*) mapping to the *msbA* gene, whose product is required for flipping LPS from the inner leaflet of IM to its outer leaflet, suggests that the absence of LapD further retards LPS translocation across the IM, which is already reduced when lipid A is underacylated.

To further reinforce these results, we next tested previously isolated single amino acid substitutions of *msbA* that restored the growth of strains synthesizing tetra-acylated lipid A Δ(*lpxL lpxM lpxP*) and Δ(*lpxM clsA*) to test if they could also restore the growth of Δ(*lpxM lapD*) bacteria. To achieve this goal, first, the deletion of the *clsA* gene from Δ(*lpxM clsA*) bacteria with the *msbA* sup* allele was eliminated by the introduction of a nearby *oppA*::spec marker, which is greater than 90% linked. The resulting Δ*lpxM msbA* sup* variants served as recipients to bring in the *lapD* deletion. In all cases, viable colonies were obtained; although, the transduction efficiency and colony size were variable, in contrast to the lethality of Δ(*lpxM lapD*) combination (Table 2). It should be noted that the presence of the *oppA*::spec marker does not influence the growth as Δ(*lpxM oppA*) still cannot accept a deletion of the *lapD* gene. Among various *msbA* suppressor-carrying strains in terms of viable colony size and number of transductants, the best suppression was observed when SR23711 (Δ*lpxM msbA* D498Y), SR23709 (Δ*lpxM msbA* S164C) and SR23707 (Δ*lpxM msbA* V287A) were used as recipients to bring in the *lapD* deletion (Table 2). A modest restoration of growth was also observed in strain backgrounds SR23705 (Δ*lpxM msbA* D431Y), SR23703 (Δ*lpxM msbA* M160I) and SR23701 (Δ*lpxM msbA* I177M) (Table 2). However, the colony size of transductants (although viable) with SR23699 (Δ*lpxM msbA* S120L) was smaller as compared to other *msbA* suppressor-carrying strains. It should be noted that Δ(*lpxM lapD*) is lethal in the absence of suppressor mutations mapping to the *msbA* gene. Thus, we can conclude that single amino acid substitutions that suppress the synthetic lethal phenotype of Δ(*lpxM clsA*) can also allow the growth of Δ(*lpxM lapD*) bacteria. Taken together, these results reveal an additional role of LapD in assisting MsbA-mediated LPS transport when the lipid A is either penta- or tetra-acylated. Thus, MsbA and LapD can collaborate in lipid A trafficking. This is more evident when mutant MsbA versions are examined, which are predicted to relax the carbon chain ruler or enhance ATP hydrolysis to accelerate LPS translocation (see Discussion section).

### 2.8. Absence of LapD Leads to Retention of LPS in the Inner Membrane

**Figure 10:**
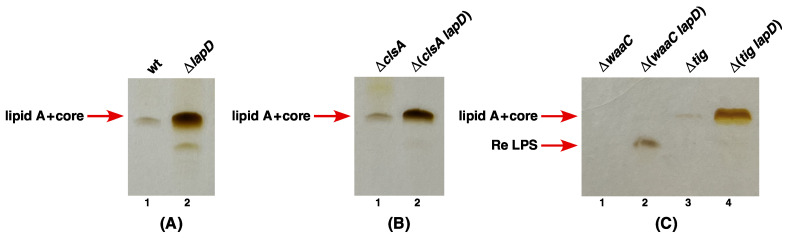
Lack of LapD causes the retention of significant amounts of LPS in the inner membrane. Total cell lysates obtained from isogenic derivatives of wild-type and Δ*lapD* bacteria were subjected to cellular fractionation to obtain the IM. Samples were treated with Proteinase K and resolved on a 16% Tricine-SDS gel. LPS was revealed by silver staining. The position of the LPS species is indicated by the arrow. Note the intense bands of LPS in the IM fraction of Δ*lapD* and its derivatives wt vs. Δ*lapD* (**A**), Δ*clsA* vs. Δ(*clsA lapD*) (**B**) and Δ*waaC*, Δ(*waaC lapD*), Δ*tig*, Δ(*tig lapD*) (**C**). The relevant genotype of strains used is indicated on the top of each panel.

As presented in the above sections, LapD co-purifies with several proteins involved in either LPS biosynthesis or its translocation. Furthermore, LapD is absolutely required for bacteria with either tetra- or penta-acylated lipid A as shown by the synthetic lethality of Δ(*lpxL lapD*) and Δ(*lpxM lapD*), respectively. Such underacylated LPS is poorly translocated by MsbA and, consistent with such results, suppressors that restore their growth were mapped to the *msbA* gene. Thus, we wondered if LPS in the absence of LapD is not efficiently translocated. To ascertain if indeed the absence of LapD results in defects in LPS translocation, isogenic cultures of wild type, Δ*lapD*, Δ*waaC*, Δ(*waaC lapD*), Δ*clsA* and Δ(*clsA lapD*) were grown at permissive temperature and shifted to 42 °C for 2 h. After the harvesting of cultures by centrifugation, total cell extracts after the removal of soluble proteins were used to obtain IM and OM fractions using sucrose gradients. Pooled fractions from the IM were treated with Proteinase K. Such samples were analyzed on a 16% Tricine-SDS gel and LPS amounts were revealed by silver staining. Such experiments clearly show that very little LPS is retained in the IM in either the wild-type (Figure 10A) or Δ*clsA* (Figure 10B) or Δ*waaC* strains (Figure 10C). However, a substantial amount of LPS was detected in the IM fraction of either Δ*lapD* or Δ(*waaC lapD*) or Δ(*clsA lapD*) bacteria (Figure 10). As an additional control, we also used LPS from IM fractions from Δ*tig* and Δ(*tig lapD*) derivatives (Figure 10C). Interestingly, a portion of LPS present in Δ*lapD* IM fractions also migrates much faster, resembling LPS of Δ*waaC* bacteria, indicating the accumulation of premature species of LPS (Figure 10A, lane 2). These results show that LapD is required for efficient translocation of LPS and, in its absence, a significant portion of LPS is retained in the IM. Moreover, the absence of LapD results in the accumulation of LPS early intermediates. Thus, quite likely, LapD and MsbA cooperate in LPS translocation.

### 2.9. Absence of LapD Causes the Constitutive Induction of LPS Defects Inducible RpoE-Dependent Stress Response

**Figure 11:**
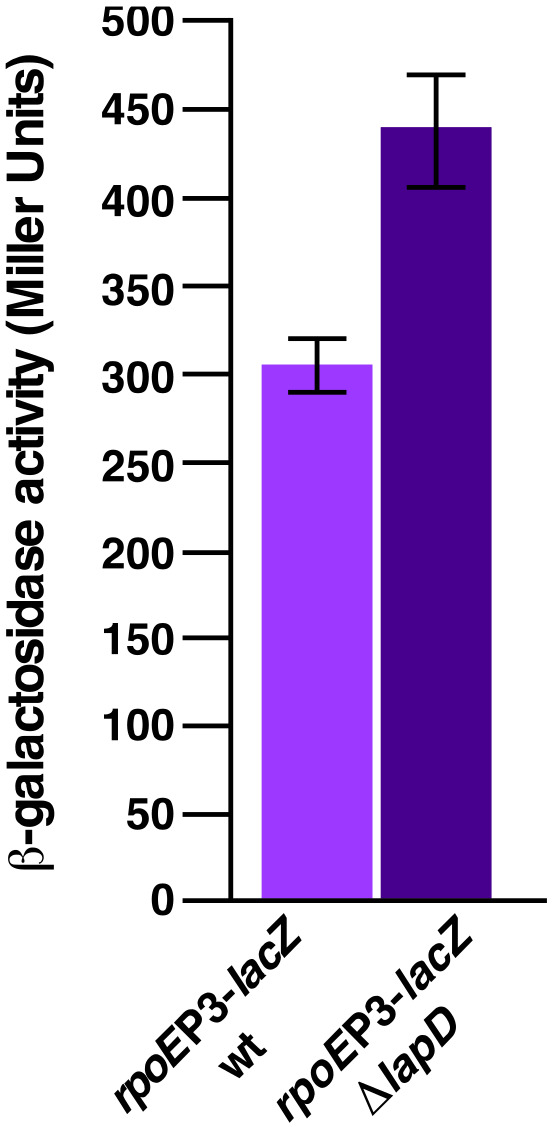
The absence of LapD causes the constitutive induction of the LPS defects responsive *rpoE*P3 promoter, even under permissive growth conditions. Exponentially grown wild type and its Δ*lapD* derivative carrying the single-copy chromosomal *rpoE*P3-*lacZ* fusion were analyzed for the *β*-galactosidase activity. Bacterial cultures were adjusted to an OD_595_ of 0.05 and allowed to grow at 30 °C. Aliquots of samples were taken to measure the *β*-galactosidase activity. Error bars represent an S.E of three independent measurements.

Previously, we have shown that severe defects in LPS biosynthesis, such as the synthesis of minimal LPS Kdo_2_-lipid IV_A_ or Δ*waaA* or Δ(*waaC lpxL lpxM lpxP*), cause a constitutive induction of RpoE-dependent cell envelope stress response [9]. A similar induction of RpoE is also observed when LPS assembly is compromised by mutations in either the *lapB* gene or the *lapC* gene [17,21]. The RpoE regulon is known to control the expression of several genes whose products are involved in either OMP maturation, folding of envelope proteins or some steps in LPS translocation and assembly [46,47,48]. It is known that the signal of LPS defects stimulates transcription of the *rpoE*P3 promoter [49]. Thus, a *lapD* deletion was transduced in the wild-type strain carrying on the chromosome single-copy *rpoE*P3-*lacZ* fusion. To measure any impact on *rpoE* transcription, the isogenic wild-type strain carrying the *rpoE*P3-*lacZ* promoter fusion and its derivative were analyzed for the *β*-galactosidase activity when cultures were grown under permissive growth conditions. Measurement of the *β*-galactosidase activity reflecting the expression of the *rpoE*P3 promoter activity showed a nearly 50% increase in strain with a deletion of the *lapD* gene under permissive growth conditions of 30 °C (Figure 11). As the *rpoE*P3 promoter activity reflects the cellular response to LPS defects, it further establishes that LapD regulates LPS assembly and its absence causes LPS defects, which in turn induces the cell envelope stress response.

### 2.10. The RpoE-Regulated MicA sRNA Is Required for the Viability of ΔlapD Bacteria

**Figure 12:**
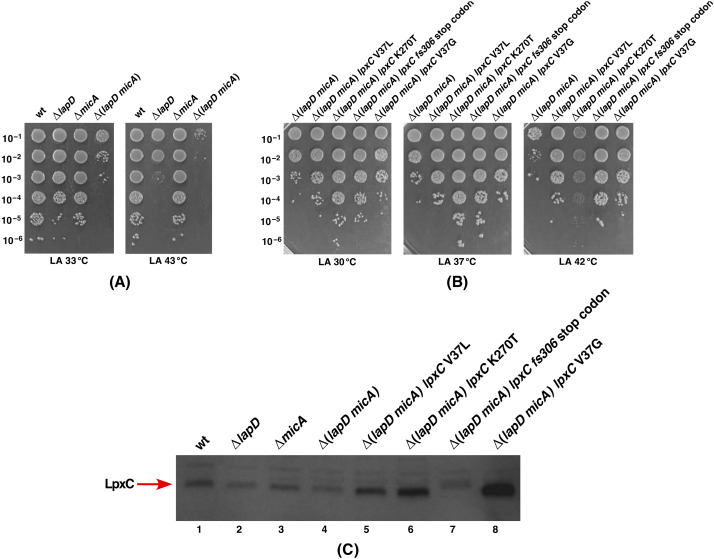
The essentiality of MicA sRNA in the Δ*lapD* background can be bypassed by mutations in the *lpxC* gene. Growth of isogenic cultures of strains of wild type, Δ*lapD*, Δ*micA* and Δ(*micA lapD*) was quantified by spot dilution on LA at 33 and 43 °C (**A**). Growth of isogenic cultures of Δ(*micA lapD*) and its derivatives carrying single amino acid mutations in the *lpxC* gene was quantified by spot dilution on LA at 30, 37 and 42 °C (**B**). An immunoblot of whole cell lysates obtained from isogenic strains with indicated genotypes using LpxC-specific antibodies. An equivalent amount of total proteins was resolved by a 12% SDS-PAGE prior to immunoblotting (**C**). Note the slower migration of LpxC-cross-reacting species in lane 7 due to a frame-shift mutation in the *lpxC* gene that adds 20 amino acids at the C-terminus. The relevant genotype and temperature of incubation are indicated.

The evidence presented so far shows that any severe compromise in LPS assembly induces the RpoE-dependent stress response and a deletion combination of *lapD* with mutations in genes whose products are involved in LPS assembly/synthesis are severely compromised for the growth. Besides investigating various null combinations, as described in Section 2.6, we also investigated if the absence of any non-essential RpoE regulon members is critical for the growth of Δ*lapD* bacteria. We specifically focused on genes encoding sRNAs whose transcription either requires the RpoE sigma factor or other sRNAs that regulate LPS modifications. Thus, several multiple deletion strain combinations with Δ*lapD* were analyzed for their growth properties. We show a specific requirement for the MicA sRNA when LapD is absent. MicA, although initially identified for its posttranscriptional repression of major OMPs such as OmpA, has also been implicated in regulating glycoform switches and is known to repress the translation of *phoP* mRNA and, hence, is linked to the regulation of LPS synthesis or its non-stoichiometric modifications [11,13,50]. MicA by itself is dispensable for bacterial growth (Figure 12A). However, significantly, for Δ(*micA lapD*) bacteria, although viable under normal growth conditions (30–33 °C), their colony size and their ability to grow were significantly impaired as determined by spot-dilution assay (Figure 12A). Moreover, Δ(*micA lapD*) bacteria are unable to propagate at temperatures above 42 °C (Figure 12A), exhibiting a synthetic lethal growth phenotype. Importantly, this synthetic lethality and severe growth defects of Δ(*micA lapD*) bacteria can be overcome when LpxC stable variants (*lpxC* V37G, *lpxC* V37L, *lpxC* K270T, and *lpxC fs306* stop codon) are introduced. For such experiments, thus SR23838 (*lpxC* V37G Δ*lapD*), SR23840 (*lpxC* V37L Δ*lapD*), SR23842 (*lpxC* K270T Δ*lapD*) and SR23844 (*lpxC fs306* stop codon Δ*lapD*) served as recipients to bring in a deletion of the *micA* gene. Viable transductants with the normal colony size were obtained at either 30 or 33 °C in all LpxC stable variant backgrounds. Comparative growth analysis of such Δ(*lapD micA*) with LpxC stable variants revealed the complete restoration of growth at either 30 or 37 °C as compared to very poor growth of a Δ(*lapD micA*) derivative (Figure 12B). Even at 42 °C, all four such derivatives with the *lpxC* mutation show the restoration of growth as compared to the lethality of a Δ(*lapD micA*) strain (Figure 12B). However, it should be noted that a (*lpxC* K270T Δ(*lapD micA*)) derivative forms relatively smaller-sized colonies at 42 °C. Thus, MicA presence is essential for the viability of Δ*lapD* bacteria, and increasing the stability of LpxC can rescue the lethal phenotype of the Δ(*lapD micA*) combination.

Next, we analyzed LpxC levels of various Δ(*lapD micA*) combinations in the presence of different *lpxC* suppressor mutations by Western blotting. Isogenic cultures of wild type, Δ*lapD*, Δ*micA*, Δ(*lapD micA*) and Δ(*lapD micA*) derivatives with *lpxC* suppressor mutations were grown at 30 °C and shifted for 2 h at 42 °C. Equivalent amounts of total proteins were resolved by SDS-PAGE and immunoblotted with LpxC-specific antibodies. The results from such an analysis clearly show that Δ(*lapD micA*) derivatives carrying the *lpxC* suppressor mutation that restore the growth at 42 °C reveal increased accumulation of LpxC (Figure 12C). This experiment again shows that Δ*lapD* bacteria have reduced amounts of LpxC (Figure 12C, lane 2). Thus, these experiments support a model wherein LapD regulates LpxC stability as Δ*lapD* bacteria have less LpxC and the main defects of such mutant bacteria stem from such a defect in regulating LpxC amounts.

### 2.11. Multicopy Suppressor Analysis to Identify Factors That Could Be Limiting When LapD Is Absent

**Figure 13:**
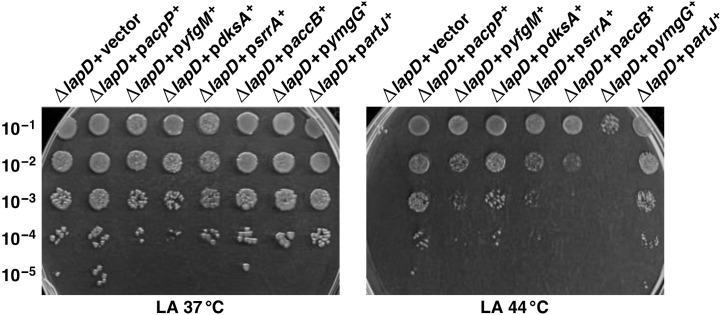
Overexpression of specific genes, including the *acpP* gene, restores the growth of Δ*lapD* bacteria at 44 °C. Growth of isogenic cultures of Δ*lapD* bacteria transformed with either plasmid DNA of the vector alone or when the specific multicopy suppressing gene is present on the plasmid was quantified by spot dilution on LA at 37 and 44 °C. The relevant genotype and temperature of incubation are indicated.

To further understand the function of LapD and the reasons for the Ts phenotype and the sensitivity towards antibiotics, such as vancomycin, when the cognate gene is absent, we employed a multicopy suppressor approach. This approach can identify genes that, when mildly overexpressed, can overcome Ts and vancomycin sensitivity and help in identifying factors that are limiting for bacterial growth when LapD is absent. Thus, we used a whole genomic library of all ORFs of *E. coli* wherein the expression of each gene is inducible from a tightly regulated P_T5_-*lac* promoter [51]. Plasmid DNA of pooled plasmids from this library was introduced into Δ*lapD* bacteria by transformation. Transformants were plated at either 44 °C or on LA medium supplemented with vancomycin (125 μg/mL) in the presence of 75 μM IPTG at 37 °C. This concentration of IPTG as an inducer of gene expression has been previously optimized with this library where the expression of most of the genes is moderate and not toxic [17,40]. Δ*lapD* transformants that grew at either 44 °C or on vancomycin-supplemented growth medium were grown to obtain plasmid DNA. Such plasmid DNA was used to retransform Δ*lapD* bacteria to ascertain the restoration of growth under non-permissive growth conditions. Validated suppressors were retained and their plasmid DNA was sequenced to identify genes whose overexpression can suppress growth defects of Δ*lapD* bacteria. This analysis identified certain genes, prominent among being *acpP*, *dksA*, *srrA*, *accB*, *yfgM*, *ymgG*, *artJ* and *artI*, which restored the growth at 44 °C (Table 3). Among these, the most robust restoration of growth at elevated temperatures was observed when the *acpP* gene is moderately overexpressed. This suppression was further verified by a spot-dilution assay in the presence of 75 mM IPTG using isogenic cultures of Δ*lapD* bacteria carrying different plasmids as compared to when only an empty vector was present. Data from such experiments show varying degrees of growth restoration at high temperature, with the nearly wild-type-like growth restoration when the *acpP* gene is present on the plasmid (Figure 13). The *acpP* gene encodes the acyl carrier protein. In *E. coli*, the acyl carrier protein (AcpP) plays a central role by sequestering and shuttling the growing acyl chain between fatty acid biosynthetic enzymes and also in providing acyl chains to LpxA, LpxD, LpxL and LpxM lipid A biosynthetic enzymes [39]. Concerning other multicopy suppressors, we previously showed that overexpression of *dksA* and *srrA* genes, which encode transcriptional factors, can restore growth at elevated temperatures when the protein folding machinery (absence of peptidyl-prolyl *cis*/*trans* isomerases) is impaired [40]. Using the same multicopy suppression approach showed that overexpression of *rcsF* and *rcsA* genes can restore resistance to vancomycin of a Δ*lapD* strain (Table 3). RcsF and RcsA belong to the two-component system that induces the expression of genes whose products are involved in colanic acid biosynthesis [52]. RcsF, located in the OM, can also sense perturbations in LPS biosynthesis and induce the signal of stress response [49,53]. Identification of AcpP as a multicopy suppressor of Δ*lapD* bacteria again reinforces the notion of the critical role played by LapD in LPS/fatty acid biosynthesis; although, which of the acceptors of AcpP are limiting requires further investigation.

### 2.12. Impact on LpxC Levels upon Overexpression of Genes That Overcome the Ts Phenotype of ΔlapD Bacteria

**Figure 14:**
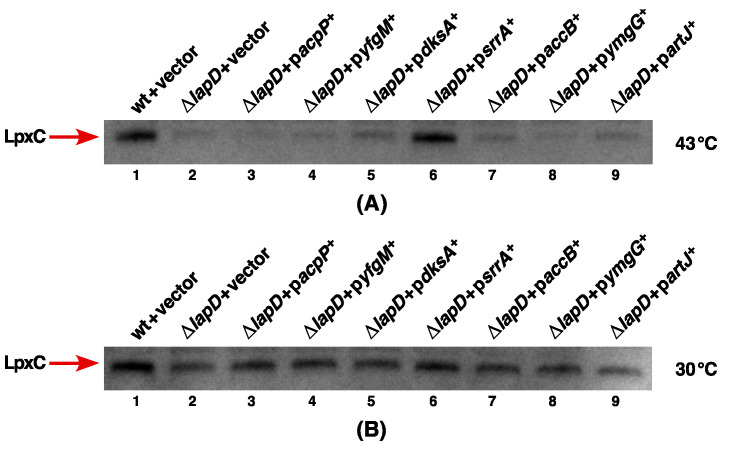
The impact of overexpression of various multicopy suppressor encoding genes in Δ*lapD* bacteria on the levels of LpxC. Immunoblots of whole cell lysates obtained from isogenic strains grown either at 30 °C, following a temperature shift for 2 h at 43 °C (**A**) or when grown at 30 °C (**B**). For immunoblotting, LpxC-specific antibodies were used and the relevant genotype is indicated. An equivalent amount of total proteins was resolved by an SDS-PAGE prior to immunoblotting. Note the restoration of LpxC levels when the *srrA* gene is overexpressed, particularly at 43 °C.

As LapD absence results in the Ts phenotype with a concomitant reduction in LpxC levels, we examined levels of LpxC by immunoblotting. Thus, using total cell extracts from the wild type and its isogenic Δ*lapD* derivatives with either an empty vector or when carrying an inducible gene whose overexpression restores the growth at high temperatures. Bacterial cultures were grown under permissive growth conditions at 30 °C and the gene expression was induced with the addition of 75 μM IPTG at OD_595_ 0.1. After 15 min of IPTG addition, an equivalent portion of each culture was shifted to 43 °C, and cultures were harvested after an additional incubation for 2 h. The equivalent amount of proteins was resolved on a 12% SDS-PAGE, and LpxC was detected by immunoblotting with LpxC-specific antibodies. At both 30 and 43 °C, LapD with the vector alone had a reduced amount of LpxC (Figure 14A, lane 2). Most significantly, only overexpression of the *srrA* gene was found to restore LpxC levels to nearly wild-type levels, particularly at 43 °C (Figure 14, lane 6). At 30 °C, also, overexpression of the *srrA* gene shows a modest increase in LpxC amounts. Surprisingly, overexpression of the *acpP* gene, which confers the best suppression at elevated temperatures, did not cause any restoration of LpxC amounts (Figure 14, lane 3). Thus, at least, we can explain that SrrA overproduction can suppress the Ts phenotype by restoring LpxC amounts. The precise function of SrrA remains unknown, except that it was also identified as a multicopy suppressor that can restore the growth of strains lacking PPIases at high temperature [40].

### 2.13. SrrA Does Not Regulate Transcription of the lpxC Gene

**Figure 15:**
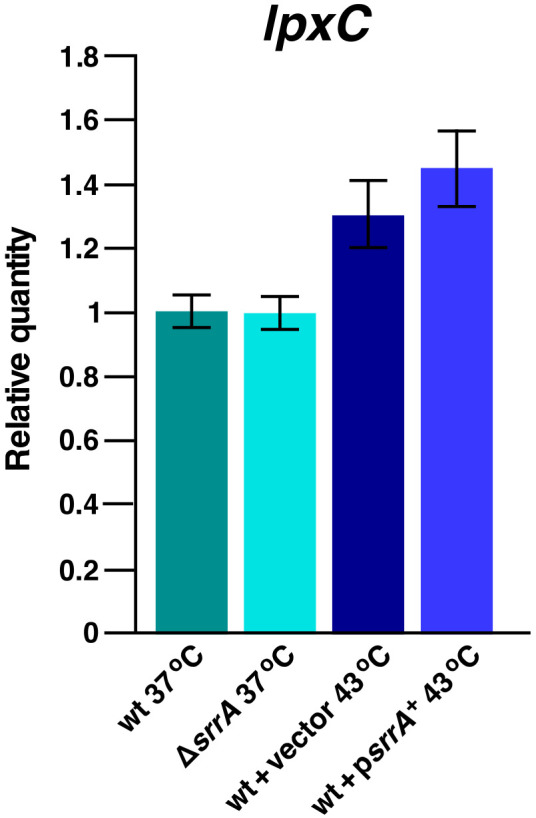
Transcription of the *lpxC* gene is not positively regulated by SrrA. q-RT-PCR analysis of mRNA extracted from wild-type bacteria, its Δ*srrA* derivative and transformed with either the vector DNA alone or when the *srrA* gene expression is induced from the IPTG-inducible promoter present in the plasmid. Isogenic bacterial cultures were grown with or without temperature shifts at the indicated temperatures. Data presented are from RNA isolated from three biological replicates and error bars are shown.

SrrA bears features of a transcriptional regulator with a conserved helix-turn-helix motif [40]. Thus, to determine if SrrA directly controls the expression of the *lpxC* gene, q-RT-PCR analysis was undertaken using gene-specific oligonucleotides for the synthesis and quantification of cDNA. For such experiments, total RNA was extracted from isogenic cultures of wild-type and Δ*srrA* bacteria grown at 37 °C. In parallel, RNA was also extracted from wild-type bacteria transformed with either the empty vector DNA alone or with plasmid DNA carrying the inducible *srrA* gene after a transient shift to 43 °C. The quantification of the *lpxC* transcription pattern showed nearly similar abundance of *lpxC* transcripts between the wild-type and Δ*srrA* bacteria (Figure 15). A shift to 43 °C showed a minor increase in *lpxC* transcripts in the wild type with the empty vector as well as when the expression of the *srrA* gene was induced (Figure 15). Thus, we can conclude that SrrA does not directly regulate *lpxC* transcription and that the increased accumulation of LpxC when SrrA is overproduced occurs at a post-transcriptional level.

### 2.14. Catalytic Activity of AcpP Is Required for Its Multicopy Suppression of Growth Defects of ΔlapD Bacteria

**Figure 16:**
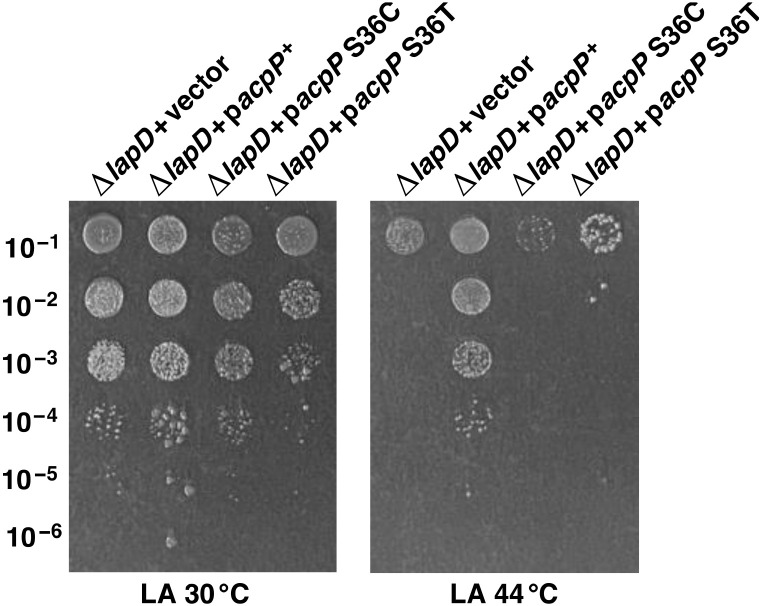
Multicopy suppression of Δ*lapD* bacteria by the *acpP* gene requires its product to retain its active site Ser 36 amino acid residue. Growth of isogenic cultures of Δ*lapD* bacteria transformed with either plasmid DNA of the vector alone or with plasmids carrying the wild-type *acpP* gene or its active site variants. The expression of the *acpP* gene is induced by the addition of 0.05% arabinose. Bacterial growth was quantified by a spot-dilution assay on LA at 30 and 44 °C. The relevant genotype and temperature of incubation are indicated.

As mentioned above, the acyl carrier protein (AcpP) plays key roles in the fatty acid and lipid A synthesis systems by mediating acyl group delivery and shuttling. ACP function requires the modification of the protein by the attachment of 4′-phosphopantetheine to a conserved Ser 36 [39,54,55]. The phosphopantetheine thiol acts to tether the starting materials and intermediates as their thioesters. Thus, in *E. coli*, AcpP is functional only in LPS and fatty acid biosynthesis after it has been posttranslationally modified by the covalent attachment of a 4′-phosphopantetheinyl (4′-PP) moiety [56]. As the *acpP* gene was identified as a multicopy suppressor of the Ts phenotype of Δ*lapD* bacteria, we tested if this suppression by AcpP requires it to be catalytically active. Thus, plasmid DNA of the pBAD24 vector containing either the *acpP* S36C gene or the *acpP* S36T gene was introduced into Δ*lapD* bacteria by transformation. In parallel, Δ*lapD* bacteria transformed with a cloned wild-type *acpP* gene or with the vector alone were used as controls. Such isogenic cultures were cultivated in the presence of glucose (0.3%) under permissive growth conditions and tested for the restoration of growth at 44 °C in the presence of 0.05% arabinose using a spot-dilution assay. The concentration of inducer arabinose was kept deliberately low since it is known that excess of ACP is toxic to bacteria. Results from such experiments reveal that the induction of expression of *acpP* S36C and *acpP* S36T cannot suppress the Ts phenotype of Δ*lapD* bacteria, while the induction of expression of the wild-type *acpP* gene can restore the growth under identical conditions (Figure 16). Results from such an experiment allow us to conclude that quite like the requirement of Ser 36 residue of AcpP in mediating acyl chain transfer in fatty acid biosynthesis, this catalytic activity is also required for AcpP to act as a dosage-dependent suppressor of Δ*lapD* bacteria. Thus, although AcpP is a very abundant protein, its increased amounts are required when LapD is absent to carry out its normal function to shuttle a growing acyl chain between biosynthetic enzymes. However, since AcpP also interacts with many other proteins that are not directly involved in the fatty acid synthesis, further experiments are required to identify the partner(s) of AcpP that are limiting in the absence of LapD.

### 2.15. LapD Is Required for Bacteria That Lack Six Major Cytoplasmic Peptidyl-Prolyl Cis/Trans Isomerases, Which Is Due to a Specific Requirement for Trigger Factor

The cytoplasm of *E. coli* contains six well-characterized peptidyl-prolyl *cis*/*trans* isomerases (PPIs), which include PpiB, Tig, SlyD, FkpB, FklB and PpiC [57]. We also recently described that DksA, Cmk and MetL exhibit the PPIase activity that can be inhibited by FK506 [40]. As DksA is a multicopy suppressor of Δ6*ppi* and also of Δ*lapD* bacteria, we examined if LapD is required when PPIs are individually or collectively absent. This was further necessitated since FklB was found to co-purify with LapD (Figure 3). Furthermore, some of the lipid A biosynthetic enzymes are known to aggregate in Δ6*ppi* strains [57]. Thus, a systemic series of bacteriophage P1-mediated transductions were executed using Δ6*ppi* bacteria as recipients. No viable transductants Δ(6*ppi lapD*) were obtained under conditions when Δ6*ppi* strains can grow (Table 4). Regarding individual PPI encoding genes, normal transductants were obtained when deletion derivatives of *ppiC*, *fkpB* or *slyD* served as recipients (Table 4). Δ(*fklB lapD*), although viable, formed smaller-sized colonies. However, severe growth defects were observed when the growth properties of Δ(*tig lapD*) were analyzed (Table 4, Figure 8). Δ(*tig lapD*) bacteria exhibited a nearly 100-fold reduction in cfu at 30 and 37 °C and with more than 10^3^-fold reduction at 42 °C. In all conditions, the colony size was severely reduced, revealing a synthetic sick phenotype of Δ(*tig lapD*) bacteria. Regarding Δ(*slyD lapD*) derivative, although viable up to 42 °C, a reduction in colony size was observed at elevated temperatures (Table 4). In contrast, no viable transductants were obtained when a *lapD* deletion was introduced in a strain lacking the *ppiB* gene. As the lethality of Δ(*ppiB lapD*) was unexpected, we reasoned that the *ppiB* deletion could be polar on the downstream essential *lpxH* gene. Consistent with such a presumption, Δ*lapD* could be readily introduced when Δ*ppiB* carrying the *lpxH* gene on a plasmid was used as a recipient (Table 4).

As DksA and Cmk exhibit a weak PPIase activity and their overproduction can restore Δ6ppi bacterial growth on rich medium at elevated temperature, we also examined their requirement. Viable transductants at a normal frequency could be obtained when a *lapD* deletion was introduced in a Δ*dksA* background at either 33 or 37 °C; however, the colony size of Δ(*dksA lapD*) bacteria is highly heterogeneous (Table 4). Significantly, Δ(*cmk lapD*) turned out to be lethal.

Taken together, we can conclude that the lethality of Δ*lapD* in Δ6*ppi* can mainly be attributed to a requirement of Tig and a deletion of the *ppiB* gene is not tolerated due to the polar effect on the expression of the downstream *lpxH* gene. Reduction in the amounts of LpxH can reduce lipid A synthesis and such results are consistent with the essentiality of LapD in strains with point mutations in genes required for the early steps of lipid A biosynthesis.

## 3. Discussion

The pivotal enzyme LpxC catalyzes the first committed step in LPS biosynthesis and the regulation of LpxC turnover is key to maintaining a balance between phospholipid and LPS biosynthesis [15,17,58]. LpxC is an unstable protein and its proteolysis is regulated by the FtsH-LapB complex [17]. This FtsH-LapB proteolysis is adjusted to match the demand for the LPS synthesis by a negative control exerted by LapC [21,22]. To fine-tune LpxC amounts, the HslVU protease complex can also degrade LpxC, which could be particularly utilized under heat shock conditions since genes encoding these proteases are regulated at the transcriptional level by the RpoH sigma factor [21]. However, we still lack complete knowledge of LpxC regulation by FtsH-LapB and LapC in terms of how they sense the LPS concentration and if they recruit any additional partners. It is also not known what are the contributions of different signals that either enhance LpxC degradation or rather render it resistant to proteolysis. Regulation of the LpxC amounts also depends on: the accumulation of precursor components of lipid A biosynthesis, levels of acyl-ACP pools, acyl-CoA, the fatty acid synthesis, growth-rate-dependent proteolysis and their individual contributions remain poorly understood [2,15,16,18,19]. In this work, we started by performing a more elaborated analysis of the LapB interactome, which revealed LapD inner membrane protein as a new additional partner that physically interacts with LapA and LapB proteins. This physical interaction was further substantiated when LapD was purified. The gene *lapD*, previously *yhcB*, was earlier identified in a screen for genes whose products are required for growth at high temperatures [34], which was again confirmed in recent studies [38]. Purification of LapD provided strong clues that LapD could be involved in LPS assembly and biosynthesis of membrane lipids since most of the LapD interactome members either participate in the LPS synthesis/transport or are involved in fatty acid biosynthesis. It needs to be emphasized that, in this study, we again observe that LapB serves as a key hub of interaction coupling LPS biosynthesis with transport. Additionally, LapB also links LpxC degradation rate with phospholipid biosynthesis since FabZ dehydratase mediating the first committed step in this pathway was also found to co-purify with FabZ. This is consistent with the previous immunoprecipitation of FabZ with LapB [17].

Besides the co-purification of LapD with LPS assembly proteins (LapA/LapB) and several proteins involved in LPS biosynthesis/transport, we carried out a systematic genetic and biochemical analysis to elaborate on the LapD function. Our data provide strong evidence that LapD plays an important role in the LPS assembly/transport and regulating LpxC amounts. This is based on: (i) An absence of LapD results in a reduction in LpxC amounts and the sensitivity towards vancomycin (membrane permeability defect). (ii) Mutations that reduce the LPS synthesis, such as *lpxA2*(ts), are synthetically lethal in Δ*lapD* bacteria. (iii) In converse, mutations that either stabilize LpxC due to mutations in the *lpxC* gene or prevent LpxC degradation (loss-of-function mutations in either the *ftsH* gene or the *lapB* gene) restore vancomycin resistance in Δ*lapD* bacteria. These very mutations in *lpxC* or *ftsH* or *lapB* genes were earlier shown to suppress the Ts phenotype of *lapC* mutants lacking its periplasmic domain and restore LpxC amounts. Thus, Δ*lapD* phenocopies a *lapC190* mutation that has a truncation of the periplasmic domain and also results in reduced amounts of LpxC. These results suggest that, quite like LapC, LapD acts upstream of LapB-FtsH in regulating LpxC levels. (iv) A deletion of the *lapD* gene is synthetically lethal with the absence of either LpxL (lauroyl acyl transferase) or LpxM (myristoyl acyl transferase). LpxL and LpxM are known to sequentially use Kdo_2_-lipid IV_A_ as a substrate to generate hexa-acylated Kdo_2_-lipid A. Critically, it is known that tetra-acylated lipid A species are selected at 1000-fold reduced efficiency by MsbA for their transport and penta-acylated lipid A derivatives could as well be transported poorly by MsbA. Thus, the synthetic lethality of Δ(*lpxL lapD*) and Δ(*lpxM lapD*) posits LapD’s involvement in LPS transport. (v) Consistent with the proposed role of LapD in LPS transport, suppressors that relieve the lethality of Δ(*lpxL lapD*) and Δ(*lpxM lapD*) bacteria map to the *msbA* gene. To strengthen the notion of LapD assisting MsbA-mediated transport of LPS, previously well-established suppressor mutations that restore the growth of strains synthesizing tetra-acylated LPS Δ(*waaC lpxL lpxM lpxP*) such as MsbA D498Y also suppress the lethality of Δ(*lpxM lapD*) bacteria. Similarly, all suppressors mapping to the *msbA* gene that relieve synthetic lethality of Δ(*lpxM clsA*) also confer the viability to Δ(*lpxM lapD*) bacteria. All such suppressor mutations are predicted to map either in the ATP-binding site of MsbA or are located in lipid A-binding/exit portals [32]. Such mutations could enhance lipid A trafficking by increasing the ATPase activity and altering the carbon chain ruler properties of MsbA, conferring a relaxed specificity to transport underacylated LPS [29,30,31,32]. (vi) Consistent with a predicted role in LPS assembly/transport, Δ*lapD* bacteria retain a substantial fraction of LPS in the IM, which is not the case when either WaaC or ClsA is absent, particularly when shifted to elevated temperatures. (vii) Deletion derivatives of *lapD* that synthesize only Kdo_2_-lipid A LPS due to lack of WaaC heptosyltransferase I are synthetically lethal at 42 °C and are very poorly tolerated even at 30 °C. The same synthetic lethal phenotype is observed when the cardiolipin synthase A encoding gene is removed in a Δ*lapD* background. Thus, defects in either early steps of LPS core biosynthesis or underacylation of lipid A and disturbance of glycerophospholipid are not tolerated when LPS assembly is impaired in the absence of LapD. However, how LapD regulates LpxC amounts via interaction with LapB needs further detailed studies, and possible mechanisms are discussed below.

Additional support for the requirement of LapD in LPS biogenesis and maintaining the cell envelope homeostasis comes from experimental evidence that Δ*lapD* bacteria exhibit a constitutive induction of the *rpoE*P3 promoter even under permissive growth conditions. Transcription of the *rpoE* gene is directed from six promoters, out of which the *rpoE*P3 promoter responds specifically to LPS defects [49]. The RpoE sigma factor regulates transcription of several genes, whose products are required for either OMP maturation (*surA*, *fkpA* and *skp*), some steps in LPS modifications (*eptB*), LPS translocation (some of the *lpt* genes), the quality control in the periplasm (*degP*) and a long operon that includes *fabZ* and *lpxD* genes [46,48,59]. The constitutive induction of the RpoE regulon could stem from LPS defects, which can also cause changes in OMP maturation. RpoE is also required for the transcription of *micA*, *rybB* and *slrA* sRNAs, constituting the non-coding repressing arm of this regulatory system [17,50]. Quite interestingly, we show that MicA sRNA becomes essential in the absence of LapD. Although a Δ(*micA lapD*) strain can be constructed, such bacteria grow extremely poorly with a small colony size in the temperature range of 30–37 °C and such bacteria are not viable at 42 °C. Since the major defect of Δ*lapD* bacteria is a reduction in LpxC amounts, the introduction of mutations in the *lpxC* gene that render encoding mutant proteins resistant to proteolysis can tolerate Δ(*micA lapD*) even at 42 °C. MicA is known to repress the synthesis of major OMPs such as OmpA and non-OMP targets such as PhoP/Q at the posttranscriptional control of gene expression [13,14,60]. A deletion of the *micA* gene in the Δ*lapD* background could thus lead to the alteration in the amounts of OMPs and relieve the repression of *phoPQ* mRNA translation. The PhoP/Q two-component system regulates lipid A modifications and also positively regulates transcription of the *mgrR* sRNA encoding gene, which represses the expression of the *eptB* gene whose product is required for the modification of the second Kdo [11,13,61]. However, how MicA absence limits LpxC amounts remains to be addressed. Thus, any major perturbation in OMP composition and in either lipid A biosynthesis or the truncation of the core region of LPS, and even potential non-stoichiometric alterations in the lipid A region of LPS are not tolerated in the absence of LapD.

While addressing the cellular requirement of LapD, we found that Δ*lapD* could not be introduced into strains lacking six known major cytoplasmic PPIases. This essentiality of LapD in the Δ6*ppi* derivative was not surprising since we have earlier shown that several enzymes involved in lipid A and phospholipid biosynthesis aggregate when all six PPIs are absent [57]. This essentiality could be attributed specifically to Tig and, to some extent, to FklB. Tig PPIase acts as a nascent chain ribosome-associated chaperone with the PPIase activity, and it has several substrates and *β*-barrel outer-membrane proteins constitute its most prominent substrates [62]. Thus, the absence of Tig could accentuate defects in OMP maturation and hence its essentiality in Δ*lapD* bacteria. As a consequence, Tig not only shows the synthetic lethality in a Δ*dnaK* or Δ*dnaKJ* background [63,64] but also in the absence of LapD. Δ(*fklB lapD*) bacteria, although viable, form small colonies, which is consistent with the co-purification of FklB with LapD. In line with such findings, overexpression of the *dksA* gene that overcomes growth defects of either Δ6*ppi* strains or a Δ*dnaKJ* derivative [40,65] was also found to suppress the Ts phenotype of Δ*lapD* bacteria. Quite interestingly, another multicopy suppressor of Δ6*ppi* bacteria, *srrA* [40], was also found to suppress the Ts phenotype of Δ*lapD* bacteria. SrrA is predicted to be a transcriptional regulator; however, genes whose expression it regulates have not been identified thus far and are currently being investigated. More related to this work, SrrA overproduction restored LpxC levels to nearly wild-type levels in Δ*lapD* bacteria, without increasing *lpxC* transcription. Thus, SrrA could regulate the expression of some genes whose products enhance either LpxC stability or prevent its degradation. Of further interest, another multicopy suppressor of Δ6*ppi*, encoded by the *cmk* gene, becomes indispensable in the Δ*lapD* background. Cytidylate kinase Cmk phosphorylates CMP and dCMP, which are produced by the turnover of CDP diglycerides and nucleic acids [66,67]. It is well established that CTP and dCTP, besides being precursors for nucleic acid synthesis, are also involved in phospholipid biosynthesis. This provides a rationale explanation for the synthetic lethal phenotype of a Δ(*lapD cmk*) combination.

Besides looking for extragenic single-copy chromosomal suppressors, we also undertook a multicopy suppressor approach that can rescue the Ts or vancomycin-sensitive phenotype of Δ*lapD* bacteria. Most interestingly, we found that a mild overexpression of acyl carrier protein encoded by the *acpP* gene can effectively restore the growth of a Δ*lapD* strain at elevated temperatures. An acyl carrier protein is a universally conserved carrier of acyl intermediates during fatty acid synthesis [39]. The major destinations of fatty acids in bacteria are glycerophospholipids, present in the IM and the inner leaflet of OM, and the lipid A part of LPS. Identification of the *acpP* gene as a multicopy suppressor of Δ*lapD* bacteria is intriguing since ACP is one of the most abundant proteins in *E. coli*, comprising nearly 0.25% of the total soluble protein [68]. Since long-chain acyl-ACPs represent only a small proportion of the total ACP pool, it is likely that in Δ*lapD* bacteria there is an alteration in the destination of acyl products, which could alter the ratio between saturated and unsaturated fatty acids. However, more studies are required to address such issues. Since the synthesis of hexa-acylated lipid A requires four ACP-dependent acyltransferases, namely, LpxA, LpxD, LpxL and LpxM, we also examined if LpxC amounts that are reduced in Δ*lapD* bacteria are altered by the induction of *acpP* gene expression. Estimation of LpxC levels did not show any restoration when the *acpP* gene was overexpressed. Despite such results, we find that for the multicopy suppression of Δ*lapD* Ts phenotype by AcpP, it is required for it to be catalytically active. This was demonstrated by mutational alteration of the active site residue Ser36 of AcpP, which is the site of prosthetic group attachment. A substitution of Ser36 by either Thr or Cys residue abrogates the suppressing ability of AcpP. Since the 4′-PP prosthetic group is attached to the hydroxyl group of a centrally located Ser36 residue by the AcpS 4′-PP transferase, any replacement of this residue results in the loss of function of AcpP in shuttling acyl chains in fatty acid biosynthesis [69]. However, it is also pertinent to point out that AcpP is known to interact with more than three dozen proteins, and all of them are not involved in fatty acid metabolism, and hence a more detailed study is required to further understand the mechanism of suppression by *acpP* overexpression. The co-purification of LapD with proteins involved in LPS and fatty acid biosynthesis pathways also identified many proteins that are also known to be part of the AcpP interactome, including LpxM, PssA and Fab enzymes. Besides these proteins, co-purifying proteins such as MukB are also known to interact with ACP [70,71]. It is likely that AcpP may also show some physical interaction with LapD and may account for some phenotypes related to cell division/chromosome segregation.

During the progression of this study, it has been suggested that LapD (YhcB) plays a role in cell division based on morphological defects and also the co-purification with some components of cell shape determination and cell division [37]. We also observed that Δ*lapD* bacteria exhibit filamentous morphology, and our own co-purification results show the interaction with ZapD, Muk proteins and MreC. Earlier studies based on the bacterial two-hybrid system have also found that LapD interacts with MreC, RodZ and LapA [35]. However, subsequent studies did not find RodZ–LapD interaction [38]. At the same time, it is important to point out that several defects in LPS biosynthesis and assembly also lead to filamentous morphology, as shown for *gmhD*, *waaC*, *lpxL* and *lapB*, (*waaC lpxL lpxM lpxP*) mutant bacteria [9,43]. Our studies do not rule out a direct link between LapD and cell division machinery; however, our suppressor approach clearly shows suppressors that either increase LpxC amounts (mutations in *lpxC*, *ftsH* and *lapB*) or enhance LPS translocation (*msbA* suppressor mutations) support direct participation of LapD in LPS assembly. Another study using whole genome transposon mutagenesis approaches also proposes that LapD (YhcB) functions at the junction of several envelope biosynthetic pathways including peptidoglycan biogenesis [36]. Some phenotypes, such as defects in biofilm formation of Δ*lapD* mutant bacteria [72], can be explained as an indirect consequence of the alteration in LPS amounts.

**Figure 17:**
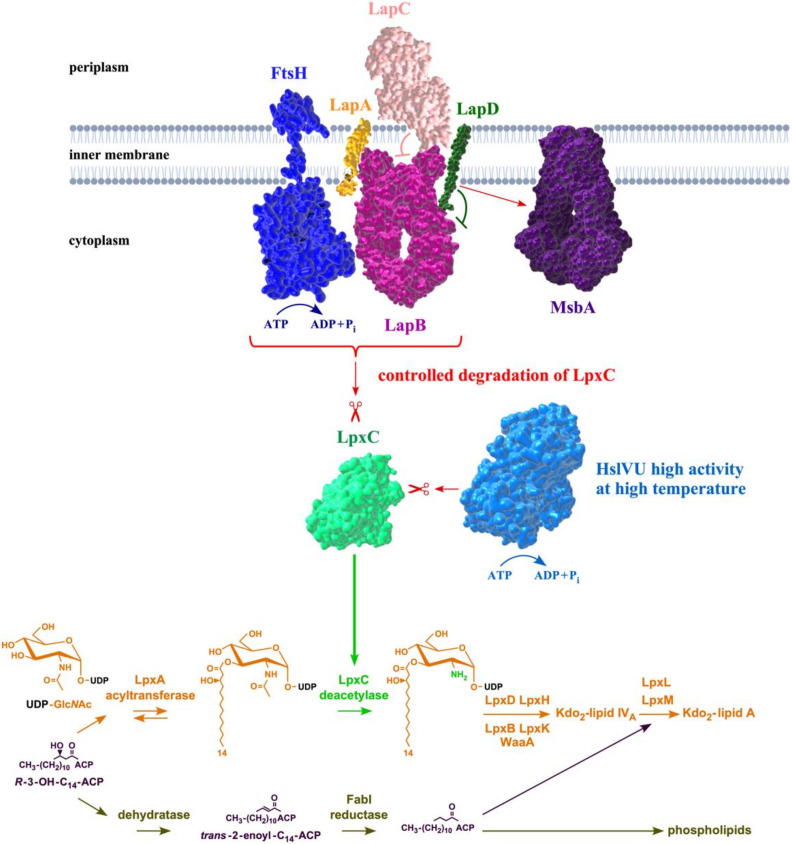
Model of LapD action in regulating LpxC levels and in assisting MsbA-mediated LPS translocation. Based on the co-purification of LapD with the LapA/LapB-FtsH complex and a reduction in LpxC amounts in the absence of LapD, it is proposed that LapD forms a complex in the IM to regulate LpxC amounts. As suppressors mapping to *lapB*, *ftsH* and *lpxC* genes restore growth defects of Δ*lapD* bacteria, LapD can act upstream of LapB as an antagonist of FtsH-mediated degradation of LpxC. This role could be similar to that earlier proposed for LapC [2]. As Δ(*lpxL lapD*) and Δ(*lpxM lapD*) are synthetically lethal and this lethality is overcome by mutations in the *msbA* gene, LapD can also assist MsbA-mediated transport of underacylated LPS species.

The model for LapD function: Based on data presented in this work, we propose that LapD functions upstream of LapB in the regulation of LpxC turnover since suppressors mapping to either the *lapB* gene (loss of function) or stable variants of LpxC that are resilient to proteolysis by FtsH overcome vancomycin sensitivity and Ts phenotype in certain combinations when LapD is absent (Figure 17). This places LapD at a junction that has so far only been assigned to LapC as an antagonist of LapB (Figure 17). All the genetic data support such a model. This model is further supported by the observed physical and genetic interactions between LapB and LapD. However, LapD function may be specifically required under conditions such as when bacteria enter a stationary phase or when OM asymmetry is compromised. In support of such a model, the stationary phase-regulated sRNA (toxin) SdsR has been shown to repress the synthesis of LapD leading to cell lysis upon its overproduction [73]. For LapD- and LapC-mediated regulation of LpxC, we need to understand some major differences. While LapC is essential, LapD is required for bacterial growth at elevated temperatures and when challenged with antibiotics such as vancomycin. The IM anchor of LapC is essential and required for the interaction with LapB, but the function of the LapD IM region could be dispensable. Although we have not addressed the requirement of the N-terminal single IM anchor of LapD, some reports find it to be dispensable [36,38], while another report suggests its requirement for LapD functionality [37]. We suggest that the LapD soluble domain could interact with the LapB cytoplasmic domain containing TPR (Tetratricopeptide Repeat) elements. Several mutations in TPR elements of LapB exhibit loss-of-function properties and structural alterations [17,21,74]. In such an interaction, LapD could act as an anti-adaptor protein, preventing excessive degradation of LpxC for proteolysis by the FtsH-LapB complex (Figure 17). In support of such a model, several loss-of-function single amino acid mutations in TPR repeats of LapB were earlier shown to suppress growth defects of *lapC* mutant bacteria [21] and in this work were shown to restore vancomycin sensitivity of Δ*lapD* bacteria as well. Alternatively, it is also possible that LapA and LapB TM regions could interact with LapD, preventing FtsH-mediated proteolysis of LpxC. Since suppressors of Δ(*lpxL lapD*) and Δ(*lpxM lapD*) map to the *msbA* gene, LapD could assist MsbA in selecting underacylated lipid A derivatives (Figure 17). However, a biochemical proof for such a LapD–MsbA interaction needs to be established. In this process of interaction with MsbA, LapC involvement is not known. LapD and cardiolipins may act similarly in assisting MsbA-mediated transport as suppressors of Δ(*lpxM clsA*) lethality mapping to the *msbA* gene also suppress the Δ(*lpxM lapD*) lethality. Consistent with such a role, Δ*lapD* bacteria retain significant amounts of LPS. This retention of LPS in the IM in Δ*lapD* bacteria makes it different from ClsA-MsbA assisting LPS transport since Δ*clsA* bacteria do not exhibit any enhanced retention of LPS, as observed in the case with the absence of LapD. Thus, in summary, we propose that LapD plays an important role in LPS assembly by regulating LpxC degradation, acting as an antagonist of LapB, and in assisting MsbA-mediated LPS translocation across the IM. As LapD is conserved in gamma-proteobacteria, this model of LpxC regulation and LPS transport could be applicable to all such bacteria in general.

## 4. Materials and Methods

### 4.1. Bacterial Strains, Plasmids and Media

The various bacterial strains and plasmids used in this study are described in Table 5. Luria-Bertani (LB) broth (Difco, Franklin Lakes, NJ, USA), M9 (Difco) and M9 minimal media were prepared as described previously [9,17]. Whenever required, growth media were supplemented with kanamycin (50 μg/mL), ampicillin (100 μg/mL), chloramphenicol (10 or 30 μg/mL), tetracycline (10 μg/mL) and vancomycin (125 μg/mL). All strains used in this study were derived from *E. coli* K-12 BW25113 strain [75], unless indicated. The construction of some of the deletion derivatives used in this study, Δ*lapB*, Δ*waaC*, Δ*tig*, Δ*dksA*, Δ*srrA*, Δ*micA*, Δ*cmk*, Δ*clsA*, Δ*lpxL*, Δ*lpxM* and *lapC190*, has been previously described [9,17,21,32,40]. A non-polar Δ*lapD* antibiotic-free deletion mutation was constructed by using the λ Red recombinase/FLP-mediated recombination system [75]. The kanamycin resistance cassette was amplified using pKD13 as a template [75]. A PCR product from such an amplification reaction was electroporated into BW25113 containing the λ Red recombinase-encoding plasmid pKD46. The *aph* cassette was removed using the plasmid pCP20 expressing FLP recombinase at either 30 °C or 33 °C. Isogenic multiple deletion combinations were constructed using bacteriophage P1-mediated transductions at either 30 or 33 °C.

### 4.2. Purification of LapD and LapB

The LapB protein was purified from solubilized IM fractions essentially as described earlier [17]. To induce the expression of the *lapD* gene, we used the minimal ORF cloned in the pCA24N expression plasmid (JW5539) [51]. In this plasmid, the expression is inducible from the P_T5_-*lac* promoter. The plasmid DNA was used to transform the wild-type strain BW25113 and the expression was induced with the addition of 300 μM IPTG at an OD_600_ 0.1 in a 1 L culture medium at 28 °C. Cultures were grown for another 5 h prior to harvesting by centrifugation at 12,000 rpm for 30 min. To obtain a relatively pure LapD protein without contamination from host proteins, the minimal coding region was cloned into the low-copy T7 promoter-based pDUET expression vector (Novagen, Warsaw, Poland) with an in-frame His_6_ tag at the N-terminus of LapD. For such experiments, the expression of the *lapD* gene was induced in BL21(DE3) derivative by the addition of 300 μM IPTG at an OD_600_ 0.1 in a 1 L culture medium at 28 °C. Cultures were further shaken till they reached an OD_600_ 0.2, followed by an addition of 200 μg/mL of rifampicin to prevent the host protein synthesis and incubated for another 2 h. Cultures were harvested by centrifugation at 12,000 rpm for 30 min at 4 °C. Pellets were frozen at −80 °C and used for further protein extraction when required. To the frozen pellet, 2X B-PER reagent (Thermo Scientific, Warsaw, Poland) was added and allowed to thaw. This mixture was adjusted to contain 50 mM NaH_2_PO_4_, 300 mM NaCl, 10 mM imidazole (buffer A), supplemented with lysozyme to a final concentration of 200 μg/mL, PMSF and a cocktail of protease inhibitors (Sigma Aldrich, Poznan, Poland) and 30 units of benzonase (Merck, Poznan, Poland). This mixture was incubated on ice for 45 min with gentle mixing. The lysate was centrifuged at 45,000× *g* for 90 min at 4 °C and pellets containing IM and OM proteins were retained. LapA/B and LapD proteins were extracted using 2% octyl-*β*-d-glucoside for solubilization of IM proteins in buffer A supplemented by PMSF and a cocktail of protease inhibitors. Solubilized IM proteins were applied over nickel-nitrilotriacetic acid beads (Qiagen, Geneva, Switzerland) and Lap proteins eluted with a linear gradient (50–500 mM) of imidazole in the presence of octyl-*β*-d-glucoside. Eluting protein fractions were analyzed by resolving on a 12% SDS-PAGE. The identity of co-eluting proteins was obtained by MALDI-TOF.

### 4.3. Immunoblotting to Estimate Amounts of LpxC

The isogenic bacterial culture of wild type, Δ*lapD* with the vector alone, and its isogenic derivatives carrying multicopy suppressor encoding genes were grown in LB medium at 30 °C, adjusted to an OD_595_ of 0.05 and allowed further growth up to an OD_595_ of 0.2. To induce the expression of the suppressing gene, IPTG at the final concentration of 75 μM was added and shifted in prewarmed flasks held at 42 °C. Cultures were harvested by centrifugation and pellets were resuspended in sample buffer. For estimating LpxC levels in the Δ(*micA lapD*) derivative with and without the presence of extragenic suppressors mapping to the *lpxC* gene, isogenic cultures were grown in LB medium at 30 °C, adjusted to an OD_595_ of 0.05 and allowed to grow for another 90 min, followed by shifting to 42 °C for another 2 h. Cultures were harvested by centrifugation. Equivalent amounts of proteins were applied to a 12% SDS-PAGE and transferred by Western blotting. Blots were probed with polyclonal antibodies against LpxC, as described previously [21]. Blots were revealed by a chemiluminescence kit from Thermo Scientific as per manufacturer’s instructions.

### 4.4. Identification of Multicopy Suppressors Whose Overexpression Suppresses Temperature and Vancomycin Sensitivity of ΔlapD Bacteria

A multicopy suppressor approach to identify either limiting factors in Δ*lapD* bacteria or find additional proteins with a function in the same pathway was essentially as previously described [76] with the following modification. The complete genomic library of all predicted ORFs of *E. coli* cloned in pCA24N [51] was used to transform Δ*lapD* strain SR23678. Transformants were plated at 44 °C on LA medium in the presence of 75 μM IPTG. In parallel, transformants were also plated on LA medium supplemented by 125 μg/mL of vancomycin at 37 °C in the presence of 75 μM IPTG. Obtained temperature-resistant or vancomycin-resistant colonies were retained. Bacterial cultures were grown from such suppressing clones and used to retransform Δ*lapD* strain SR23678 to verify the suppression. DNA insert of all relevant plasmids that yielded reproducible results was sequenced to obtain the identity of the multicopy suppressing gene.

### 4.5. Introduction of Various Suppressor Mutations Mapping to lpxC, lapB, ftsH and msbA in ΔlapD and Its Derivatives

We previously described the isolation of extragenic suppressors of GK6075 with a Cm cassette replacing the entire periplasmic domain of the LapC (*lapC190*) strain [21]. Such single amino acid substitutions mapped to either *lpxC* or *lapB* or *ftsH* genes restored the growth at elevated temperatures and suppressed permeability defects. To test if such suppressor mutations can also overcome permeability defects (vancomycin sensitivity), the *lapC190* mutation was replaced by a wild-type copy of the *lapC* gene by bringing in a closely linked marker using bacteriophage P1-mediated transduction. Thus, a bacteriophage P1 was grown on a strain (SR9710) carrying a *napA*::Tn*10* insertion (70% linked to the *lapC* gene) with an intact wild-type *lapC* gene and used as a donor selecting for Tet resistance with strains SR22731, SR22738, SR22727, GK6098 and GK6094 serving as recipients (Table 5). All such recipient strains contain the *lapC190*::cm mutation and a single amino acid substitution in the *lpxC* gene (Table 5). Tet^R^ colonies that lost the Cm cassette were retained. A representative strain from each transduction Tet^R^ Cm^S^ was first verified to have retained the *lpxC* suppressor mutation with the wild-type copy of the *lapC* gene by DNA sequence analysis of PCR products using specific oligonucleotides to amplify coding regions of *lpxC* and *lapC* genes. After such verification, one strain each with a different *lpxC* suppressor mutation (SR23812, SR23814, SR23816, SR23818 and SR23820, Table 5) served as a recipient to bring in Δ*lapD* Kan^R^ replacement. Transductants were plated at 33 °C and analyzed further. The same strategy of replacing the chromosomal *lapC190* mutation with the wild-type of the *lapC* gene carrying a suppressing mutation mapping to either the *ftsH* gene (GK6095) or in the *lapB* gene (SR22724, SR22726, SR22730, SR22733, and GK6084) using *napA*::Tn*10* as a donor in transductions was adopted. This was followed by introducing the *lapD* null allele.

Concerning testing the suppression of lethality of Δ(*lpxM lapD*), *msbA* suppression mutations that overcome the lethality of Δ(*lpxM clsA*) combination were used for such a reconstruction. To achieve this, previously constructed strains SR23302, SR23303, SR23305, SR23309, SR23313, SR23315 and SR23316 [32], all carrying different single amino acid substitutions in the *msbA* gene with a chromosomal deletion combination of *lpxM* and *clsA* genes, served as recipients to first replace the deletion of the *clsA* gene by the wild-type copy of this gene. To achieve this, a bacteriophage P1 lysate was grown on strain SR23138 with an *oppA*::*ada* mutation, which served as a donor for the above-mentioned strains with Δ(*lpxM clsA*) msbA* combinations. The *oppA* gene is more than 90% linked to the *clsA* gene. Thus, Spec^R^ transductants were selected and those that were Kan^S^ (replacement of Δ*clsA* by the wild-type copy) were retained. The presence of a specific *msbA* suppressor was verified by PCR amplification. The resulting strains then served as recipients to introduce a deletion of the *lapD* gene in the Δ*lpxM* background.

### 4.6. Isolation of Suppressor Mutations That Confer Viability to Δ(lpxL lapD) and Δ(lpxM lapD) Derivatives and Their Mapping

As Δ(*lpxL lapD*) and Δ(*lpxM lapD*) combinations turned out to be lethal, we sought suppressor mutations that allow their growth. Towards this goal, multiple rounds of transductions were executed in Δ*lpxL* and Δ*lpxM* backgrounds to bring in a deletion of the *lapD* gene. Transductants were plated on LA medium at 30 °C and incubated for 72 h. Surviving transductants were streak purified and one strain from each combination was retained. Chromosomal DNA from such strains SR23684 Δ(*lpxL lapD*) and SR23685 Δ(*lpxM lapD*) was used as a template to amplify several candidate genes that included *lpxC*, *lapA*, *lapB*, *ftsH*, *fabZ* and *msbA*. As both of them had a different single-amino acid substitution in the *msbA*, we ruled out the presence of any additional mutation by replacement of msbA* with the wild-type copy using a linked marker.

### 4.7. RNA Purification and q-RT-PCR Analysis

Exponentially grown isogenic cultures of wild type and its Δ*srrA* derivative, and strains carrying the inducible *srrA* gene present on a plasmid were grown at 37 °C in LB medium, adjusted to an OD_595_ of 0.05 and allowed to further grow up to an OD_595_ of 0.2. In the case of strains with either a vector alone or when the *srrA* gene was present on the plasmid, 75 μM IPTG was added prior to the shift up of temperature. For heat shock, aliquots were shifted to prewarmed medium held at 43 °C and incubated for 15 min. Total RNA was purified by hot phenol extraction as described [77]. Purified RNA was treated with RQI RNase-free DNase (Promega, Madison, WI, USA) to remove any chromosomal DNA, and RNA was ethanol precipitated and resuspended in DEPC-treated water. RNA amounts were quantified and their integrity verified by agarose gel electrophoresis. q-RT-PCR was used to quantify changes in the *lpxC* gene expression in Δ*srrA* and the wild type and when the expression of the *srrA* gene was induced, using gene-specific primers. Purified mRNA (2 μg) was converted to cDNA using Maxima H-Minus Reverse Transcriptase (Thermo Scientific). Reactions were carried out for 40 cycles using PowerUp SYBR^®^ Green PCR Master Mix (Thermo Scientific), as described previously [57]. q-RT-PCR was performed using the CFX Connect Real-Time PCR Detection System (Bio-Rad, Warsaw, Poland). Data were analyzed by software Bio-Rad CFX Maestro.

### 4.8. Separation of Inner and Outer Membranes to Quantify LPS

Isogenic cultures of the wild type, its Δ*lapD*, Δ(*waaC lapD*), Δ*clsA* and Δ(*clsA lapD*) derivatives were grown under permissive growth conditions (LB 30 °C) up to an OD_595_ 0.8. Cultures were harvested by centrifugation and cells were broken by French Press. Unbroken cells were removed by centrifugation at 3500 rpm for 15 min. The total cell lysate was subjected to centrifugation at 20,000 rpm for 90 min to remove soluble proteins and the membrane fraction resuspended in 1 mM Tris-HCl, pH 7.5, 20% sucrose. Samples were applied to a two-step sucrose gradient. The IM and the OM were separated by ultracentrifugation at 23,000 rpm for 18 h at 4 °C using an SW28 rotor (Beckman, Warsaw, Poland). The IM fractions located between 20% and 53% sucrose were pooled, treated with Proteinase K for 2 h and resolved on a 16% Tricine-SDS. LPS was visualized by silver staining.

### 4.9. Growth Analysis and Measurement of β-galactosidase Activity

For the quantification of bacterial growth and measurement of sensitivity to vancomycin, exponentially grown cultures were adjusted to an optical density OD_595_ of 0.1. Samples were prepared using ten-fold dilutions and analyzed by spot-dilution assay on agar plates at different temperatures or when supplemented by 125 μg/mL of vancomycin. An amount of 5 μl of each dilution was spotted on agar plates and bacterial growth analyzed after incubation for 18–24 h at indicated temperatures. To measure the impact on the envelope stress response, isogenic cultures of the wild type and its *lapD* deletion derivative carrying the *rpoE*P3-*lacZ* promoter fusion were grown at 30 °C. Cultures were adjusted to an optical density OD_595_ of 0.05 and allowed to grow at 30 °C for another 45 min. Aliquots of cultures were taken after different time intervals of growth and analyzed for *β*-galactosidase activity as described previously [49]. For each assay, three independent cultures were used and the average of each was plotted.

## Figures and Tables

**Table 1:** A Δ*lapD* mutation is lethal when the LPS synthesis is impaired, which is the opposite in the case of Δ*lapB* strains.

Genotype	Number of Transductants		Viability in Δ*lapD*
	P1 Δ*lapD* LA 30 °C	P1 Δ*lapB* M9 30 °C	
wt	953	96 small colonies	viable
*lpxA2*(ts)	12 sc ^1^	650	not viable
*lpxB1*(ts)	9	439	not viable
*lapC190*	17 sc	475	not viable

^1^ sc indicates small colony size.

**Table 2:** Suppressor mutations mapping to the *msbA* gene can rescue the synthetic lethality of Δ(*lpxM lapD*) bacteria as judged by their viability.

Genotype	P1 Δ*lapD* Number of Transductants LA 30 °C	Viability and Colony Size
wt	1243	viable
Δ*lpxM*	6	not viable
Δ*lpxL*	13	not viable
Δ(*lpxM lapD*) *msbA* S120L	620	viable small size
Δ(*lpxM lapD*) *msbA* M160I	786	viable medium size
Δ(*lpxM lapD*) *msbA* I177M	730	viable small size
Δ(*lpxM lapD*) *msbA* Y287A	1490	viable normal size
Δ(*lpxM lapD*) *msbA* D431Y	735	viable normal size
Δ(*lpxM lapD*) *msbA* S165C	1130	viable normal size
Δ(*lpxM lapD*) *msbA* D498Y	1267	viable normal size
Δ*waaC*	433	viable small size not viable at 42 °C
Δ*clsA*	378	viable small size not viable at 42 °C

**Table 3:** Identification of the most relevant multicopy suppressors of Δ*lapD* strains.

Name	Number of Transformants		Function
	LA 44 °C	vancomycin	
vector alone	9 small colonies	6 small colonies	
*acpP*	750	ND ^1^	acyl carrier protein
*dksA*	486	ND	transcriptional regulator
*srrA*	520	ND	transcriptional regulator
*yfgM*	430	ND	inner membrane protein, substrate of FtsH
*accB*	511	ND	acetylocoenzyme carboxylase
*rcsF*	ND	701 medium size colonies	colanic acid regulator
*artJ*	473	ND	L-arginine ABC transporter

^1^ ND denotes not determined since suppressors were isolated either at 44 °C or on vancomycin-supplemented growth medium at 37 °C.

**Table 4:** Requirement of LapD in strains lacking cytoplasmic PPIases.

Name	P1 Δ*lapD* Number of Transductants LA 33 °C	Features
wt	1300	viable
Δ6*ppi*	5	not viable
Δ*ppiB*	9	not viable
Δ*ppiB* + p*lpxH*^+^	920	viable
Δ*ppiC*	1430	viable
Δ*tig*	230 sc ^1^	conditional lethality at high temperatures
Δ*fklB*	490 sc	viable but smaller colony size
Δ*fkpB*	735	viable
Δ*slyD*	506	viable but colony size reduced above 42 °C

^1^ sc indicates small colony size.

**Table 5:** Bacterial strains and plasmids used in this study.

**Strains**	**Genotype**	**Reference**
BW25113	*lacI*^q^*rrnB*_T14_ Δ*lacZ*_WJ16_ *hsdR514* Δ*araBAD*_AH33_ Δ*rhaBAD*_LD78_	[75]
SR23678	BW25113 *lapD*<>*aph*	This study
SR23743	BW25113 *lapD*<>*frt*	This study
SR17532	BW25113 φ(*rpoEP3*-*lacZ*)	[9]
SR23686	SR17532 *lapD*<>*aph*	This study
SR8233	W3110 *waaC*<>*cat*	[9]
SR23691	SR23678 *waaC*<>*cat*	This study
SR23320	BW25113 *clsA*<>*frt*	This study
SR23769	SR23320 *lapD*<>*aph*	This study
SR8522	W3110 *micA*<>*cat*	[11]
SR23852	BW25113 *micA*<>*cat*	This study
SR23900	SR23678 *micA*<>*cat*	This study
SR21068	BW25113 *tig*<>*cat*	[57]
SR23773	SR21068 *lapD*<>*aph*	This study
SR23684	Δ(*lpxL lapD*) *msbA* L412P	This study
SR23685	Δ(*lpxM lapD*) *msbA* V287A	This study
SR23699	BW25113 *lpxM msbA* S120L	This study
SR23701	BW25113 *lpxM msbA* I177M	This study
SR23703	BW25113 *lpxM msbA* M160I	This study
SR23705	BW25113 *lpxM msbA* D431Y	This study
SR23707	BW25113 *lpxM msbA* V287A	This study
SR23709	BW25113 *lpxM msbA* S164C	This study
SR23711	BW25113 *lpxM msbA* D498Y	This study
GK6075	BW25113 *lapC190*	[21]
GK6078	*lapC190 lpxC* K270T	[21]
GK6094	*lapC190 lpxC fs306* stop codon	[21]
SR22727	*lapC190 lpxC* R230C	[21]
SR22731	*lapC190 lpxC* V37G	[21]
SR22738	*lapC190 lpxC* V37L	[21]
GK6095	*lapC190 ftsH* A296V	[21]
SR22724	*lapC190 lapB* H325P	[21]
SR22726	*lapC190 lapB* A88V	[21]
SR22730	*lapC190 lapB* H181R	[21]
SR22733	*lapC190 lapB* R115H	[21]
GK6084	*lapC190 lapB* D124Y	[21]
GK6087	*lapC190 lapB* R125L	[21]
SR23812	*lpxC* R230C	This study
SR23814	*lpxC* V37G	This study
SR23816	*lpxC* V37L	This study
SR23818	*lpxC* K270T	This study
SR23820	*lpxC fs306* stop codon	This study
SR23822	*ftsH* A296V	This study
SR23836	*lpxC* R230C *lapD*<>*aph*	This study
SR23838	*lpxC* V37G *lapD*<>*aph*	This study
SR23840	*lpxC* V37L *lapD*<>*aph*	This study
SR23842	*lpxC* K270T *lapD*<>*aph*	This study
SR23844	*lpxC fs306* stop codon *lapD*<>*aph*	This study
SR23905	SR23840 *micA*<>*cat*	This study
SR23907	SR23842 *micA*<>*cat*	This study
SR23909	SR23844 *micA*<>*cat*	This study
SR23911	SR23838 *micA*<>*cat*	This study
SR23846	*ftsH* A296V *lapD*<>*aph*	This study
SR23857	*lapB* H325P *lapD*<>*aph*	This study
SR23859	*lapB* A88V *lapD*<>*aph*	This study
SR23861	*lapB* H181R *lapD*<>*aph*	This study
SR23863	*lapB* R115H *lapD*<>*aph*	This study
SR23865	*lapB* D124Y *lapD*<>*aph*	This study
SR23867	*lapB* R125L *lapD*<>*aph*	This study
SR22995	SR20491 Δ(*lapA lapB*)	This study
SR23138	BW25113 *oppA*<>*ada*	This study
SM101	*lpxA2*(ts)	CGSC, Yale
MN7	*lpxB1*(ts)	CGSC, Yale
SR23798	SR23678 + pCA24N	This study
SR23790	SR23678 + p*acpP*^+^	This study
SR23784	SR23678 + p*yfgM*^+^	This study
SR23786	SR23678 + p*dksA*^+^	This study
SR23792	SR23678 + p*srrA*^+^	This study
SR23796	SR23678 + p*accD*^+^	This study
SR23794	SR23678 + p*ymgG*^+^	This study
SR23788	SR23678 + p*artJ*^+^	This study
SR23729	SR23678 + pEB540 pBAD-CBP-ACP	This study
SR23732	SR23678 + pEB547 pBAD-CBP-ACP (S36C)	This study
SR23735	SR23678 + pEB797 pBAD-CBP-ACP (S36T)	This study
SR23738	SR23678 + pBAD24	This study
**Plasmids**	**Genotype**	**Reference**
pCA24N	IPTG-inducible expression vector cm^R^	[51]
pDUET	expression vector	Our collection
pKD3	*oriR6K_g_*, *bla*(Amp^R^), *kan*, *rgnB*(Ter), *cat*	[75]
pKD13	*oriR6K_g_*, *bla*(Amp^R^), *kan*, *rgnB*(Ter)	[75]
pKD46	*araBp*-*gam*-*bet*-*exo*, *bla*(Amp^R^), *repA101*(ts)	[75]
pCP20	ts replicon with inducible FLP recombinase	[75]
JW5539	*lapD*^+^ in pCA24N	[51]
pSR23599	*lapD*^+^ in pDUET	This study
pSR23790	*acpP*^+^ in pCA24N	This study
pEB540	pBAD-CBP-ACP	[55]
pEB547	pBAD-CBP-ACP (S36C)	[55]
pEB797	pBAD-CBP-ACP (S36T)	[55]

## Data Availability

Data are contained within the article.
